# Unveiling Therapeutic Powers of Indigenous Flora: Antimicrobial, Antioxidant, and Anticancer Properties of *Horwoodia dicksoniae*

**DOI:** 10.3390/ph18050765

**Published:** 2025-05-21

**Authors:** Khadijah A. Altammar

**Affiliations:** Department of Biology, College of Science, University of Hafr Al Batin, P.O. Box 1803, Hafr Al Batin 31991, Saudi Arabia; khadijahaa@uhb.edu.sa

**Keywords:** *Horwoodia dicksoniae*, *Stipa capensis*, anticancer, antimicrobial, antioxidant, chemical composition, GC-MS, LC-MS/MS, molecular docking

## Abstract

**Background:** *Horwoodia dicksoniae* Turrill. (Brassicaceae) and *Stipa capensis* Thunb. (Poaceae) are commonly grown in the eastern region of Saudi Arabia. **Methods**: This study evaluated the antibacterial and antifungal potential of these plants. *H. dicksoniae* extract was further subjected to antioxidant, anticancer, GC-MS, LC-MS/MS, and in silico analyses. **Results**: *H. dicksoniae* extract presented a higher antimicrobial efficiency than *S. capensis* extract by effectively inhibiting the growth of *Staphylococcus aureus*, *Escherichia coli*, *Proteus vulgaris*, *Bacillus subtilis*, and *Candida albicans*. *H. dicksoniae* ethanolic extract also demonstrated promising antioxidant and anticancer properties against the human colon cancer cell line HCT-116. GC-MS analysis revealed the presence of 12 natural compounds in the *H. dicksoniae* extract, whereas LC-MS/MS analysis revealed 19 different compounds in negative ion mode and 25 in positive ion mode. Furthermore, the presence of bioactive compounds in the *H. dicksoniae* extract, such as flavonoids (acacetin and hesperetin) and caffeic acid, confirmed the observed antibacterial, antifungal, antioxidant, and anticancer activities. Molecular docking revealed promising interactions between various bioactive compounds and target proteins associated with antimicrobial, antioxidant, and anticancer activities. **Conclusions**: This study is the first to report GC-MS and LC-MS/MS analyses of *H. dicksoniae* ethanolic extract. The findings provide valuable insights into the potential mechanisms and therapeutic applications of the identified bioactive compounds. Thus, the present work can serve as a platform for the isolation of natural compounds from *H. dicksoniae* extract, which may play a significant role in the discovery and design of new drugs for the treatment of human diseases.

## 1. Introduction

The pharmaceutical and biochemical properties of medicinal plants can be harnessed for the prevention and treatment of various diseases. Plants are an important source of natural antioxidants and therapeutic agents, which can be used to develop novel medicines. Medicinal plants have been employed as therapeutic agents since ancient times, and their antimalarial, antioxidant, antitumor, antidiabetic, anti-analgesic, anti-inflammation, antimicrobial, and antiviral properties further enhance their importance in the current global scenario [1,2,3,4,5]. Saudi Arabian flora is rich in medicinal plants (>2250 species) and a high proportion (24.57%) are used in folk medicines [6,7]. *H. dicksoniae* (known in Arabic as Khuzama), belonging to the Lepidieae tribe of the Brassicaceae family, and *S. capensis* (Poaceae) naturally grow in the Kingdom of Saudi Arabia. In this study, plant samples (*H. dicksoniae* and *S. capensis*) were collected from the Al-Samman region in the northeastern region of the country. Brassicaceae (Cruciferae or the mustard family) is a major angiosperm family, which comprises 4636 species across 340 genera [8]. Poaceae (grass family) is a major cosmopolitan family comprising 11,000 flowering plant species, which grow on all continents and in all types of climatic zones. This family has significant medical, pastoral, and economic importance [9].

The aerial parts of *H. dicksoniae* contain various chemical compounds, including 1-feruloyl-β-D-glucopyranoside and tryptophane methyl ester. The medicinal efficacy of these compounds is well documented, as they can effectively restrict the growth of *Aspergillus fumigatus* and human cancer cells (breast, liver, and lung cancers) [7]. *H. dicksoniae* extract can efficiently inhibit and terminate the growth of *Streptococcus pneumoniae* and *Escherichia coli* as well [10]. The aerial parts of *H. dicksoniae* contain triterpenes, flavonoids, sterols, and glucosinolates, whereas its volatile oil makes it a rare aromatic plant of Brassicaceae. The flavonoid compounds of *H. dicksoniae* mainly include apigenin 6-C-β-D-galactopyranoside, luteolin-7-O-β-D-glucopyranoside, and luteolin 6-C-β-D-galactopyranoside [11]. Luteolin (3′,4′,5,7-tetrahydroxyflavone) and luteolin-7-O-β-D-glucopyranoside are known to exert antitumor and cytotoxic impacts on human cancer cell lines. Luteolin can selectively restrict and inhibit the growth of colon cancer cells [12,13,14]. Moreover, Fawzy et al. [11] reported that *H. dicksoniae* extract attenuated renal dysfunction and malondialdehyde (MDA) levels, which restored oxidant balance and enhanced the activity of renal antioxidant enzymes and non-enzymatic glutathione (GSH).

In this study, native *H. dicksoniae* and *S. capensis* plants in Saudi Arabia were investigated for their antibacterial and antifungal properties. The extract of the more efficient plant species (*H. dicksoniae*) was further tested against a colon cancer cell line, and its antioxidant activity was assessed by measuring its radical-scavenging activity. Gas chromatography–mass spectrometry (GC-MS) and Liquid chromatography–tandem mass spectrometry (LC-MS/MS) analyses of *H. dicksoniae* extract were performed for chemical characterization. Furthermore, the computational molecular docking technique was employed to elaborate on the underlying bioactivity-related mechanisms and depict the binding interactions of the identified *H. dicksoniae* bioactive compounds with key target proteins in essential microbial pathways, as well as anticancer and antioxidant target proteins. Collectively, this study explored the bioactive and therapeutic potential of the indigenous Saudi Arabian *H. dicksoniae* plant.

## 2. Results

### 2.1. Antifungal and Antibacterial Activities of Horwoodia dicksoniae and Stipa capensis Extracts

Table 1 presents the antifungal and antibacterial activities of ethanolic extracts of *H. dicksoniae* and *S. capensis*. The *H. dicksoniae* extract demonstrated the highest antifungal activity against *C. albicans*, with a maximum inhibition zone of 20.6 ± 1.5 mm, which was significantly higher than the inhibition zone of the *S. capensis* extract (16.3 ± 0.5 mm). The ethanolic extracts of both plants did not exhibit any antifungal activity against *A. fumigatus*. The *H. dicksoniae* extract exhibited higher antibacterial efficacy against Gram-positive bacteria such as *S. aureus* and *B. subtilis*, with the diameter of the inhibition zone being 18.6 and 15 mm, respectively. The *H. dicksoniae* extract resulted in the largest inhibition zone of 22.4 mm in diameter against Gram-negative *E. coli*, whereas the inhibition zone of *P. vulgaris* had a diameter of 8 mm. The inhibition zones of the *S. capensis* extract had a comparatively smaller size with a diameter of 17.8, 13, and 21 mm against *S. aureus*, *B. subtilis*, and *E. coli*, respectively. However, this extract did not show any activity against *P. vulgaris*.

### 2.2. Minimum Inhibitory Concentration (MIC)

Table 2 depicts the MIC values of the *H. dicksoniae* and *S. capensis* extracts against *C. albicans*, *S. aureus*, *B. subtilis*, *E. coli*, and *P. vulgaris*. The MIC values of the *H. dicksoniae* and *S. capensis* extracts against *C. albicans* were noted as 1.95 and 31.25 μg/mL, respectively. The MIC values of both plant extracts against pathogenic bacteria ranged between 0.98 and 12.81 μg/mL. *H. dicksoniae* exhibited higher activity against *S. aureus*, *B. subtilis*, *E. coli*, and *P. vulgaris*, with lower MIC values of 0.98, 7.81, 1.95, and 3.90 μg/mL, respectively. The MIC values of the *S. capensis* extract against *S. aureus, B. subtilis,* and *E. coli* were noted as 7.81, 12.81, and 5.60 μg/mL, respectively, and this extract did not demonstrate any activity against *P. vulgaris*.

### 2.3. Total Antioxidant and Radical-Scavenging Activities of H. dicksoniae Extract

**Figure 1:**
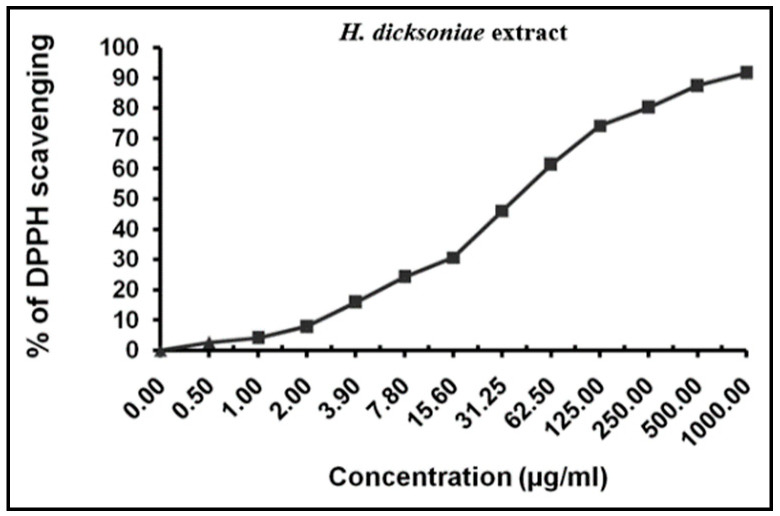
Antioxidant activity of *H*. *dicksoniae* ethanolic extract using 2,2-diphenyl-1-picrylhydrazyl (DPPH). Values are presented as means ± SE (n = 3).

The better-performing *H. dicksoniae* extract with higher antifungal and antibacterial activities was subjected to further evaluation. The *H. dicksoniae* extract’s antioxidant activity was estimated in terms of IC_50_ value, which is the value at which 50% of free DPPH radicals are scavenged. A high antioxidant potential is denoted by a smaller IC_50_ value for the identified plant constituents. An IC_50_ value of 39.26 ± 3.04 µg/mL was noted for the *H. dicksoniae* extract under the experimental conditions (Figure 1), whereas an IC_50_ value of 10.62 ± 0.84 µg/mL was found for ascorbic acid. The IC_50_ value of the *H. dicksoniae* extract indicates its ability to prevent oxidation and resist free radicals.

### 2.4. MTT Assay for Cytotoxicity Analysis

**Figure 2:**
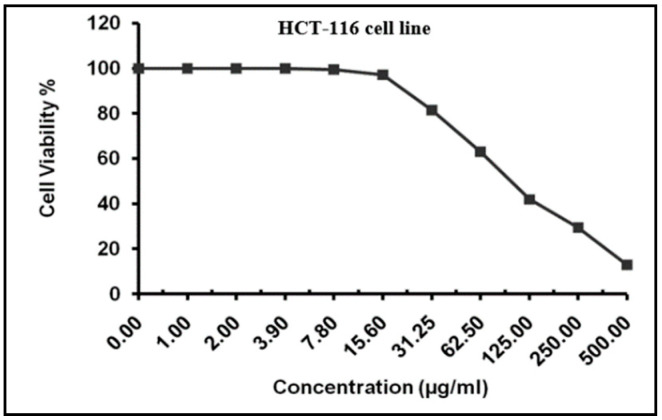
Anticancer activity of *H*. *dicksoniae* ethanolic extract against HCT-116 cell line. Values are presented as means ± SE (n = 3).

The MTT assay revealed the cytotoxic effects of different *H. dicksoniae* concentrations on HCT-116 cells (a human colon cancer cell line) after 24 h. The IC_50_ value of the extract was noted as 101.12 ± 5.49 µg/mL against HCT-116 cells (Figure 2).

### 2.5. GC-MS Analysis of H. dicksoniae Ethanolic Extract

**Figure 3:**
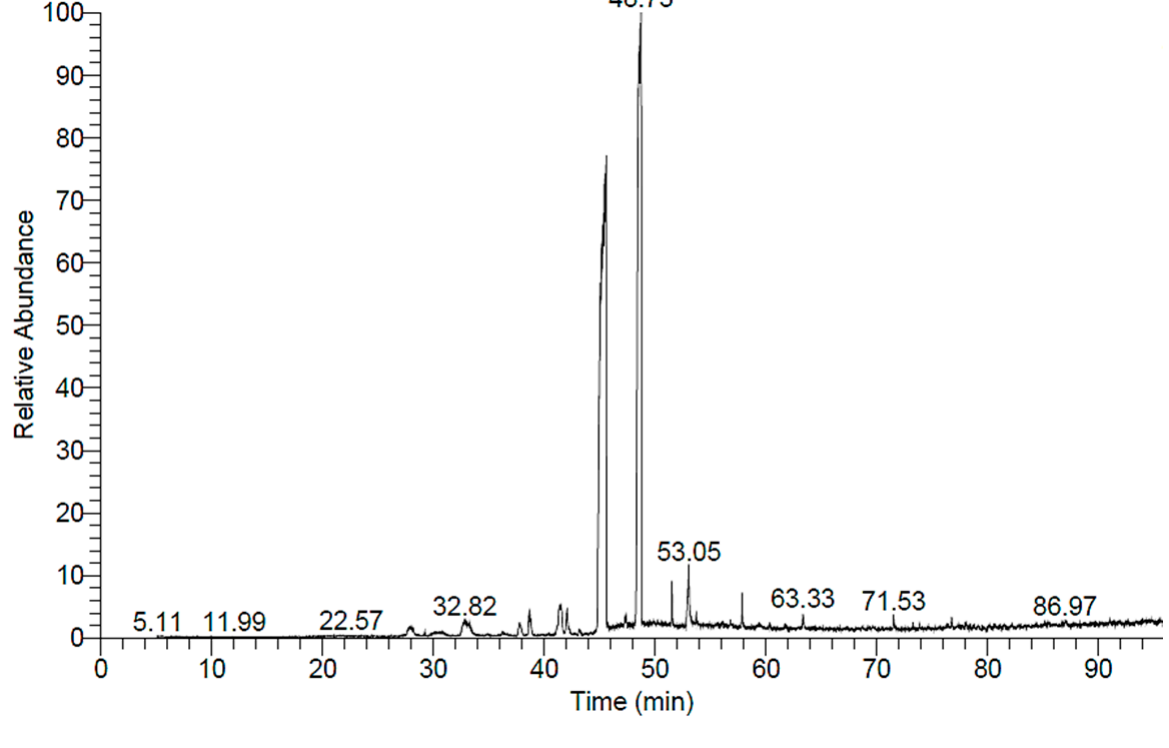
GC–MS based compound profile of *H. dicksoniae* ethanolic extract.

GC-MS analysis was conducted, and the spectrum is depicted in Figure 3. A total of 12 compounds were identified, and these compounds are summarized in Table 3. The mass spectrum and chemical structures of the compounds identified in the ethanolic extract of *H. dicksoniae* are shown in Figure S1 (Supplementary Materials). Isopropyl myristate (also known as tetradecanoic acid, 1-methylethyl ester) (44.53%) was found to be a major compound, followed by benzoic acid, phenylmethyl ester (14.31%), and n-Hexadecanoic acid (2.29%). The percentage compositions of the remaining 12 constituents ranged from 0.14% to 1.01%.

### 2.6. LC-MS/MS

LC-MS/MS was employed to profile the bioactive ethyl acetate extract of *H. dicksoniae* that was fractioned from the 70% ethanol extract. The negative and positive ionization modes were adopted using the ReSpect database for phytochemicals. Table S1 (Supplementary Materials) summarizes the identified compounds in both the positive (25) and negative (19) ionization modes. It also presents the retention time (RT), formula, precursor ion, error, adduct, reference, and MS/MS. A total of 6 out of 44 different compounds were found in both positive and negative ionization modes, including caffeic acid, luteolin-3′,7-di-O-glucoside, luteolin-6-C-glucoside, apigenin 8-C-glucoside, luteolin, and 3,5,7-trihydroxy-4′-methoxyflavone. The identified metabolites belong to various classes of natural products (see Supplementary Table S1).

**Figure 4:**
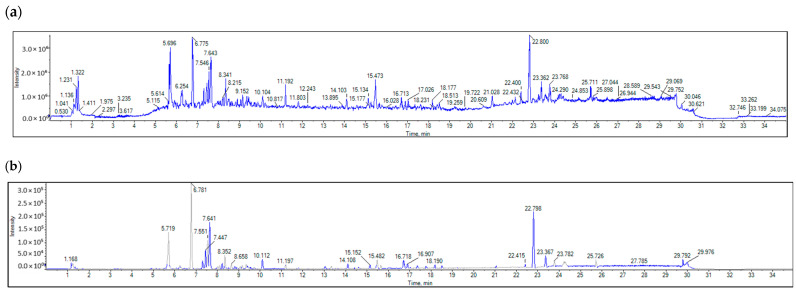
Positive ESI-MS spectra; (**a**) total ion chromatogram (TIC), (**b**) base peak chromatogram (BPC) of the plant extract in positive mode. The peak values represent retention time (min).

**Figure 5:**
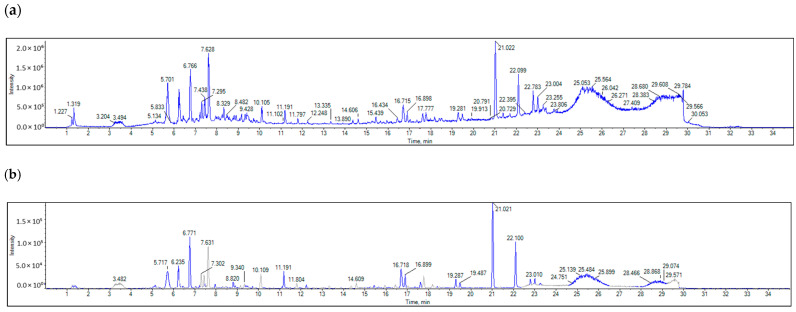
Negative ESI-MS spectra; (**a**) total ion chromatogram (TIC), (**b**) base peak chromatogram (BPC) of the plant extract in negative mode. The peak values represent retention time (min).

The positive and negative ESI-MS spectra (Figure 4 and Figure 5) reveal the presence of aniline and substituted anilines (4-aminophenol), nicotinamides, three alpha-amino acids (L-arginine, pipecolate, and L-norleucine), aminopurines (adenine), cholines, proline, O-glycosyl compounds (Melibiose), hydroxycinnamic acids (caffeic acid), hydroxycinnamic acid glycosides (1-O-b-D-glucopyranosyl sinapate), four flavonoid-O-glycosides (luteolin-3′,7-di-O-glucoside, kaempferol-7-neohesperidoside, quercitrin, and myricitrin), two flavonoid-C-glycosides (luteolin-6-C-glucoside and apigenin-8-C-glucoside), flavonoid-3-O-glucuronides (kaempferol-3-glucuronide), two 4′-O-methylated flavonoids [acacetin (5,7-dihydroxy-4′-methoxyflavone) and hesperetin (4′-methoxy-5,7,3′-trihydroxyflavanone)], flavones [luteolin (3,4,5,7-tetrahydroxyflavone)], flavonols (3,5,7-trihydroxy-4′-methoxyflavone), five anthocyanidin-O-glycosides [cyanidin-3-O-(2″-O-beta-glucopyranosyl-beta-glucopyranoside), cyanidin-3-glucoside, peonidine-3-O-glucoside chloride, cyanidin-3-O-galactoside, and cyanidin-3-O-rutinoside], aurone O-glycosides (maritimetin-6-O-glucoside), 6-methoxy-7-hydroxycoumarin (scopoletin), dihydroxycoumarins (daphnetin), indoles (3-formylindole), monosaccharide phosphates (alpha-D-glucose-1-phosphate dipotassium salt dihydrate), purine ribonucleoside monophosphates (inosine-5′-monophosphate), trihydroxy bile acids, alcohols and derivatives (cholic acid), catechins ((+)-3,3′,4′,5,7-pentahydroxyflavan), Linoleic acid and derivatives (gamma-linolenic acid), sugar acids and derivatives (L-threonic acid hemicalcium salt), and hydropyridines (isoguvacine).

### 2.7. Molecular Docking of Antimicrobial, Anticancer, and Antioxidant Target Proteins with Phytochemical Compounds (Ligands)

A detailed understanding of the protein–ligand interactions is central to understanding biology at the molecular level. Moreover, knowledge of the mechanisms responsible for the protein–ligand recognition and binding, and understanding the effects of these compounds on proteins that are directly involved in the development of diseases, will pave the way for the detection of new precursor compounds as well as facilitating the discovery, design, and development of new drugs. In our study, molecular docking investigation was conducted to model the interactions between some bioactive GC-MS- and LC-MS/MS-identified compounds of *H. dicksoniae* and target proteins (receptor) associated with antimicrobial, antioxidant, and anticancer activities. Table 4 (showing only the lowest binding energy that the ligand needs to bind to the proteins) and Supplementary Table S2 (showing the binding energy of all analyzed ligands and the target proteins) present the binding affinity (∆G)-based interaction strength between target proteins and GC-MS-identified ligands. Favorable ligand–protein binding interactions are represented by negative ∆G values. A higher negative ∆G value indicates the binding affinity is enhanced. As shown in Table 4, three compounds (ligands) showed a high binding affinity with the tested proteins. The anticancer proteins CA2 and PRKCE showed the highest affinity with benzoic acid, phenylmethyl ester. However, PPARA showed enhanced affinity with hexadecanoic acid, phenylmethyl ester. For the three antioxidant target proteins, FABP2 showed the highest affinity towards hexadecanoic acid, phenylmethyl ester, whilst FABP3 and FABP4 displayed the highest affinity towards benzoic acid, phenylmethyl ester and 1-(4-isopropylphenyl)-2-methylpropyl acetate, respectively. For antimicrobial targets, benzoic acid, phenylmethyl ester exhibited the higher affinity towards most fungal (ERG2, ERG5, and ERG11) and bacterial targets (rpsQ-*E. coli*, dacC-*B. subtilis*, and pbp-*S. aureus*). However, ERG3 and rpsA-*P. vulgaris* showed a high affinity towards 1-(4-isopropylphenyl)-2-methylpropyl acetate. These data would suggest that the ligand has the potential to efficiently exert its therapeutic effects through its strong binding affinity towards its target, as binding affinity is affected by various factors such as molecular shape, hydrogen bonding, electrostatic interactions, van der Waals forces, and hydrophobicity. The 2D and 3D binding interactions of favorable ligands and antibacterial, antifungal, anticancer, and antioxidant target proteins are demonstrated in Table 5 (see also Supplementary Table S3).

Likewise, Table 6 (showing only the lowest binding energy that the ligand needs to bind to the target proteins) and Supplementary Table S4 (showing the binding energy of all analyzed ligands and the target proteins) show the binding affinity (∆G)-based interaction strength between a number of LC-MS-identified ligands and target proteins. As shown in Table 6, only eight compounds (ligands) showed a high binding affinity with the tested proteins. Interestingly, the anticancer target proteins—CA2 and PRKCE—and PPARA proteins showed high affinity with kaempferol-7-neohesperidoside and luteolin-3′,7-di-O-glucoside, respectively. The antioxidant proteins FABP2, FABP3, and FABP4 showed a high binding affinity with kaempferol-3glucuronide, apigenin 8-C-glucoside, and cyanidin-3-O-rutinoside, respectively. Similarly, the fungal protein ERG3 showed a high affinity with three ligands, namely, acacetin, hesperetin, and 3′,5,7-trihydroxy-4-methoxyflavanone. However, ERG2 showed a high affinity to acacetin, whereas ERG5 and ERG11 showed a high affinity to luteolin-3′,7-di-O-glucoside. Finally, cyanidin-3-O-rutinoside showed the highest affinity to dacC of *B. subtilis* and rpsQ of *E. coli*, whereas luteolin-3′,7-di-O-glucoside showed the highest affinity to rpsA of *P. vulgaris* and pbp of *S. aureus*. Table 7 (see also Supplementary Table S5) displays the 2D and 3D binding interactions with the highest negative ∆G values between the LC-MS-identified ligands and target proteins. In particular, several conventional hydrogen and carbon–hydrogen bonds were observed. Other non-covalent interactions of the Pi–cation, Pi–anion, Pi–Pi T-stacked, Pi–alkyl, Pi–sulphur, Pi–sigma, Pi–donor hydrogen bond, and amide–Pi stacked types were also observed in the majority of the studied compounds.

## 3. Discussion

Antimicrobial resistance has emerged as a global threat to animal and human health [15]. The WHO published a priority list of fungal pathogens in 2022, whereas the initial priority list of bacterial pathogens was published in 2017 and updated in 2024 [16]. The WHO periodically evaluates antifungal and antibacterial agents to devise priorities and facilitate research developments. Plant compounds rich in antimicrobial and antifungal bioactive agents have attracted researchers’ interest for the development of novel antibiotics [17]. Thousands of plant species have undergone in vitro investigations, establishing the antibacterial (Gram-negative and Gram-positive) and antifungal potential of several plant extracts and pure compounds [18]. Plants’ activities are mainly attributed to their alkaloid and flavonoid contents [19,20,21].

In this study, the antimicrobial investigation revealed the effectiveness of *H. dicksoniae* and *S. capensis* extracts. Plant extracts with MIC values < 8 mg/mL are known to possess antimicrobial properties. However, extracts with MIC values < 100 µg/mL generally possess significant antibacterial potential, with an MIC range of 100 to 625 µg/mL considered moderate and a value > 625 µg/mL considered low [18]. In this study, the MIC values of the tested extracts (0.98 µg/mL to 12.81 µg/mL) indicated their significant antibacterial potential. However, these MIC values were lower than the standards (gentamicin, ampicillin, and amphotericin B). The MIC values demonstrated the higher antibacterial and antifungal efficacy of the *H. dicksoniae* extract than that of *S. capensis*.

Neither of the plant extracts inhibited *A. fumigatus* growth; however, the ethanolic extract of *H. dicksoniae* demonstrated higher antifungal and antibacterial potential than that of *S. capensis*. Abdelwahab et al. [7] reported the inhibitory effects of *H. dicksoniae* methanolic extract against *Syncephalastrum racemosum*, *A. fumigates*, *Geotricum candidum*, *Streptococcus pneumoniae*, *E. coli*, and *B. subtilis*, but they did not notice any activity against *C. albicans* and *P. aeruginosa.* The different compositions of methanolic, ethanolic, and aqueous plant extracts could impact their antimicrobial spectrum [7,22]. Moreover, the inhibitory efficacy of different plants’ ethanolic extracts could vary with different microorganisms. This could be due to their varying phytochemical compositions and modes of action [19,23]. *C. albicans* exhibited more sensitivity than *A. fumigatus*, whereas Gram-positive bacteria demonstrated lower sensitivity compared to *E. coli*, a Gram-negative bacterium. The different cell wall permeability of fungi and bacteria could have contributed to this phenomenon. A Gram-positive bacterial cell wall contains teichoic acids and peptidoglycan (murein), whereas a Gram-negative bacterial cell wall is composed of lipoproteins and lipopolysaccharides [24]. The fungal cell wall is mainly composed of glucan and chitin polysaccharides [25]. Thus, the cell wall permeability varies in these microorganisms [26]. A study by Darah et al. [27] revealed that an antimicrobial compound’s interaction with the cell membrane is the main mechanism behind its antibacterial properties. Sahgal et al. [28] linked differences in the MIC values to cell morphology and composition. The antimicrobial activity of plant extracts could be associated with the acidic hydroxyl group on the aromatic ring of plant phenolic compounds [22,29]. The lipophilic properties of these compounds facilitate their penetration into the plasma membrane to cause ionic homeostatic disturbance. Moreover, hydroxyl groups inhibit phosphorylation reactions and electron transport. Plant phenolic compounds are particularly known to affect fungal growth-regulating enzymes [10].

Plant extracts are generally rich in secondary metabolites; however, their antimicrobial activity does not solely depend on these metabolites. Other factors such as concentration and interaction among different components could also play a crucial role in their antimicrobial efficacy [30]. Plant alkaloids intercalate with bacterial DNA and interfere with cell division to exert antibacterial effects [31]. The flavonoids in plant extracts act against microorganisms by binding with intracellular soluble proteins and the cell walls of bacteria, whereas steroids form complexes with membrane lipids to cause cell leakage [19]. The antimicrobial properties of plant saponins are based on the leakage of important enzymes and proteins from the microbial cell [32].

The results of this study indicated a higher antimicrobial potential of *H. dicksoniae* ethanolic extract compared to that of *S. capensis*. Therefore, the pharmacological traits of *H. dicksoniae* were further analyzed in this study. Although data on the bioactive compounds of *S. capensis* are scarce or completely absent, some species of the *Stippa* genus such as *S. tenacissima* have demonstrated to be a promising source of pharmacological and biological activities (showing antioxidant properties and antiproliferative effects against HT-29 cell lines) [33]; the full range of biological activities will be covered in a separate (unpublished) research paper. The ethanolic extract of *H. dicksoniae* exhibited free radical-scavenging and antioxidant activities and, thus, could be considered a natural source of antioxidants. Abdelwahab et al. [7] reported the high antioxidant potential of *H. dicksoniae* methanolic extract. To the best of our knowledge, this study is the first to report the antioxidant activity of *H. dicksoniae* ethanolic extract. Antioxidant activity could vary with the use of different solvents. For example, Akthar et al. [34] demonstrated the antioxidant activity of *Adenium obesum* extracts in different solvents to be in the order of chloroform > water > methanol > butanol > ethyl acetate > hexane. Similarly, Mehmood et al. [35] reported the highest antioxidant properties in the chloroform extract of *Geranium pusillum* leaves. Free radicals are linked to various diseases, including diabetes, artery blockage, cancer, and anti-inflammatory process [36]. Phenolic and flavonoid compounds of plants are known for their higher radical-scavenging and antioxidant potential [34]. Butylated hydroxyamisole (BHA), tert-butylhydroxytoluene (TBHQ), butylated hydroxytoluene (BHT), and propylgallate (PG) are common synthetic antioxidants. However, these antioxidants have been reported to cause carcinogenesis and liver damage in experimental animals [37]. Therefore, the development of safe natural antioxidants is crucial.

Cancer is the second-leading cause of global mortality after heart disease [38]. Recent studies have established the pharmacological efficiency of plant bioactive compounds against various malignancies [39]. In this study, the MTT assay depicted an IC_50_ value of 101.12 ± 5.49 µg/mL against the HCT-116 cell line for the *H. dicksoniae* extract. Abdelwahab et al. [7] also reported that *H. dicksoniae* exhibited cytotoxicity against A-549 cells (human lung carcinoma cells), MCF-7 cells (human breast cancer cells), and HepG-2 cells (human liver cancer cells), with IC_50_ values of 43.3, 37.0, and 38.5 µg/mL, respectively. Merajuddin et al. [40] demonstrated a moderate anticancer capability of *A. monosperma* with a cell viability range of 11.9 to 16.7%. Several studies have established the anticancer properties of various plants, which could differ with various organic solvents. Khan et al. [41] reported the high cytotoxicity of *Chenopodium glaucum (n*-hexane and methanolic extracts) against human lung carcinoma cells. Notably, the results of this study revealed a promising anticancer potential of *H. dicksoniae* ethanolic extract, which requires further in-depth investigations. To the best of our knowledge, the anticancer effectiveness of *H. dicksoniae* ethanolic extract has never been reported before. Therefore, a detailed phytochemical analysis of *H. dicksoniae* ethanolic extract was performed, which could lead to the identification of potential phyto-molecules as therapeutic agents from *H. dicksoniae*, a plant grown abundantly in Saudi Arabia.

GC-MS and LC-MS/MS analyses of *H. dicksoniae* ethanolic extract were performed to identify the major bioactive molecules. This study is the first to report GC-MS and LC-MS/MS analyses of *H. dicksoniae* ethanolic extract; therefore, the results were compared with other medicinal plants. The GC-MS analysis revealed the presence of different fatty acids and their derivatives. The major components were isopropyl myristate (also known as tetradecanoic acid, 1-methylethyl ester) followed by benzoic acid, phenylmethyl ester. Smaller amounts of other compounds were also detected, which might have contributed to the extract’s biological activity via their interaction with various proteins. Lazzeri et al. [42] reported that the oil content in Brassicaceae seeds could range from 10 to 45% of dry matter, which explains the high numbers and percentages of fatty acids. The varying fatty acid compositions of plant oils confer different biological properties for the production of bioenergy. Moreover, polyunsaturated fatty acids are beneficial for human health [43,44]. Fatty acids play a major role in plants’ antifungal and antibacterial activities [45]. Generally, algae and plants produce fatty acids for protection against pathogens and multidrug-resistant bacteria. The OH groups of fatty acids affect bacterial cell membranes through their amphipathic property, which facilitates the solubilization of various membrane components, including lipid bilayers and proteins, to cause cell lysis. Different fatty acids such as hexadecanoic acid, pentadecanoic acid, methyl ester, 14-methyl-, methyl ester, 3,4,4-Trimethyl-5-pyrazolone, and 1,2-Benzenedicarboxylic acid are well known for their antifungal and antimicrobial activities [46]. Shaaban et al. [47] conducted GC-MS analysis of clove alcoholic extract and identified an active compound known as hexadecenoic acid methyl ester (found in *H. dicksoniae* extract) with the highest antimicrobial effect against clinically pathogenic bacteria. Isopropyl myristate is well known as a solvent, emulsifier, and moisturizer with polar characteristics commonly used in cosmetics and topical medical preparations to ameliorate skin absorption. Pakki et al. [48] investigated the effectiveness of isopropyl myristate as a penetration enhancer agent to augment the diffusion rate of antioxidant creams containing extract from kasumba turate seeds (*Carthamus tinctorius* L.). The antibacterial properties of isopropyl myristate found in *H. dicksoniae extract* have been reported when used as a solvent in sterility testing [49]. Moreover, previous literature surveys revealed that phthalic acid esters (PAEs) were detected in different parts of 60 plant species that belong to 38 families, as well as those isolated and purified from various algae, bacteria, and fungi. The dominant PAEs identified from natural sources generally include di-n-butyl phthalate, diethyl phthalate (identified in *H. dicksoniae* extract), dimethyl phthalate, di(2-ethylhexyl) phthalate, diisobutyl phthalate, and diisooctyl phthalate, among others, which have been reported to possess allelopathic, antimicrobial, insecticidal, and other biological activities. PAEs were also found in Brassicaceae (*Brassica oleracea*) [50]. Fatty acid composition and fat quantity are key factors in the growth of colon tumors. Unsaturated fatty acid exerts significant cytotoxicity in various diseases [43,44]. Similarly, the biological activities (nematicide, anti-inflammatory activity, antifungal, hypocholesterolemic, and abenzointibacterial) of hexadecanoic acid found in *H. dicksoniae* extract have been previously reported as well [51,52]. The anticancer, hepatoprotective, antioxidant, hypocholesterolemic, and antimicrobial properties of 9-octadecenoic acid have been established in several studies [52,53,54]. In addition, the volatile oil extracted from the roots of *Cynanchum stauntonii* exhibited activity against influenza virus in vitro, as 2(3H)-furanone, dihydro-5-pentyl (detected in *H. dicksoniae* extract) was found to be one of the major components in the oil when it was examined using gas GC-MS [55].

The organic and aqueous extracts of Brassicaceae plants have been established to be rich sources of tocopherols, phenolics, ascorbic acid, flavonoids, and carotenoids, advocating for their pharmaceutical effectiveness [56]. Mattosinhos et al. [57] also reported that the secondary metabolites of Brassicaceae plants, including glycosylates, terpenes/carotenoids, and polyphenols, contribute to the antioxidant, healing, and anti-inflammatory effects. Sohaib et al. [29] reported varying activities of plant extracts according to the plant parts and habitats. They further revealed phenols as the major constituents of ethanolic extracts from *Morenga oleifera*, *Avicennia marina*, and *Phragmites australis*. Moreover, the antioxidant activities demonstrated a positive correlation with phenolic contents and played a key role in cytotoxic effects. *M. oleifera*’s leaves were particularly active against the HepG2 cell line, as they had comparatively higher phenolic contents.

Phenolic compounds are subdivided into subcategories, including simple phenols, phenolic acids, coumarins, flavonoids, tannins, stilbenes, lignans, quinones, and curcuminoids; they exhibit antimicrobial, anti-inflammatory, antiviral, hepatoprotective, anticarcinogenic, antiallergic, and antioxidant actions. Thus, phenolics are considered to be potential therapeutic agents against diabetes, cancer, cardiovascular dysfunctions, neurodegenerative diseases, inflammatory diseases, and anti-aging [58]. The presence of benzoic acid, classified as a phenolic substance, is part of the chemical defense mechanism of plants against microbes. It shows antimicrobial properties and the ability to inhibit the growth of various microorganisms, including bacteria, fungi, and yeast, by disrupting the pH balance within microbial cells [59]. Notably, molecular docking studies of bioactive compounds revealed that some phenolic substances showed organism-specific binding preferences. For example, benzoic acid, phenylmethyl ester demonstrated favorable binding interactions with a majority of microbial target proteins. Likewise, it showed a favorable binding affinity towards target proteins associated with anticancer and antioxidant activities. This result highlighted the importance of examining specific interactions when determining binding affinity and specificity. Hydrophobic non-covalent (pi-stacking, pi-alkyl and pi-sigma) interactions and hydrogen bonds were identified as the key facilitators of ligand–protein binding, and these interactions contribute to the overall binding strength and determine the specificity of ligands for their target proteins. This specificity could be exploited for the development of targeted antimicrobial, anticancer, and antioxidant agents.

Moreover, LC-MS/MS analysis detected several bioactive compounds, especially flavonoids. Flavonoids (phenolic compounds) are commonly found in seeds, plants, and fruits. These secondary metabolites give them distinctive fragrance, color, and flavor. Flavonoids (hesperetin and acacetin) positively affect human health through their neuroprotective, anti-inflammatory, immunomodulatory, anticancer, cardioprotective, anti-aging, antidiabetic, antiviral, antibacterial, and antiparasitic properties [20,60]. Interestingly, this study also detected efficient antifungal potential, particularly against *C. albicans*. Acacetin and hesperetin demonstrated strong binding affinities (−8.7 kcal/mol) with the ERG3 protein of *C. albicans*. ERG3 is crucial for the biosynthesis of ergosterol, which is a key component of fungal cell membranes. Hydrophobic, pi–cation, and hydrogen-bonding interactions could disrupt ERG3 function to affect the integrity of fungal cell membranes. This mechanism explains these compounds’ antifungal activity against *C. albicans*. It is known that acacetin is an O-methylated flavone found in various dietary sources and plants that possesses vasorelaxant, antimicrobial, anti-obesity, anti-inflammatory, antimalarial, anticancer, and antioxidant characteristics [21]. Hesperetin is also a natural flavanone glycoside with antioxidant and anti-inflammatory properties and plays a role in the inhibition of tumor cell metastasis and angiogenesis; its efficacy against insulin resistance has been proven using in vivo and in vitro models. Thus, it could be an effective treatment to tackle diabetes and its side effects. Hesperetin is also known to prevent the occurrence of ferroptosis, conferring protecting against intervertebral disc degeneration [61].

In addition, this study showed that the interaction between luteolin-3′,7-di-O-glucoside and the target protein PPARA (peroxisome proliferator-activated receptor) associated with anticancer activity was prominent, with a −9.2 kcal/mol binding affinity. PPARA regulates lipid metabolism to exert anticancer and anti-inflammatory impacts [62]. It forms multiple hydrogen bonds with residues such as SER280, CYS276, and ALA333 to achieve stable binding. Pi–sigma interactions with LEU321 and THR279 further stabilize the binding. This interaction exerts beneficial impacts on inflammation and lipid metabolism by modulating PPARA activity. Likewise, the luteolin-3′,7-di-O-glucoside ligand exhibited the highest binding affinity with target proteins (ERG5 and ERG11 of *C. albicans*, *P. vulgaris*, and *S. aureus*) associated with antimicrobial activities. This finding is in accordance with other studies reporting that luteolin effectively prevented pulmonary and hepatic fibrosis and reduced cancer cell proliferation. Interestingly, this compound can also play a protective role in inflammation and cancer by modulating the microbiota. Luteolin is among the main flavones found in food such as onion and celery [63].

The compound cyanidin-3-O-rutinoside belongs to the anthocyanin class of flavonoids that and possesses potent antioxidant, anti-inflammatory, antidiabetic, and anticancer activities [64]. The interaction between cyanidin-3-O-rutinoside and FABP4 (a fatty acid-binding protein) was quite significant, exhibiting a binding affinity of −10.4 kcal/mol. This strong interaction suggested anticancer potential, as FABP4 contributes to cancer (ovarian, colorectal, breast, and prostate) metastasis and progression [65]. The compound establishes multiple conventional hydrogen bonds with many residues to stabilize its position inside the binding pocket. Moreover, anion–pi interactions with ASP77 and GLU73 enhance its binding stability. These interactions could disrupt FABP4′s signaling and lipid transport functions to restrict cancer cell proliferation and growth. Similarly, cyanidin-3-O-rutinoside had a high binding interaction with the target proteins of *B. subtilis* (dacC) and *E. coli* (rpsQ) associated with antibacterial activity. The interaction of cyanidin-3-O-rutinoside and the dacC protein (−9.2 kcal/mol) of *B. subtilis* is particularly important for antibacterial properties. PBPs contribute to bacterial cell wall synthesis, and their inhibition is a common mode of action of beta-lactams. The compound establishes multiple hydrogen bonds with various polar and charged amino acids residues for a stable interaction, which potentially inhibits PBP function and disrupts the synthesis of bacterial cell walls.

Moreover, kaempferol-3-glucuronide and kaempferol-7-neohesperidoside, belonging to the flavonol class of flavonoids, were identified in this study, and their interactions with CA2 and PRKCE were high, exhibiting a binding affinity of −8.0 and −9.8 kcal/mol, respectively. Kaempferol, which can be found in a wide variety of herbs and plant families including Brassicaceae, is the most well-known and studied flavonoid for its anti-inflammatory, antioxidant, and antitumor properties and capability to prevent cancerous cell proliferation [66]. Kaempferol and its associated compounds also exhibit antibacterial, antifungal, and antiprotozoal activities. Several studies showed that the potential action mechanism behind the antibacterial activity of kaempferol is cell membrane disruption, followed by the activation of apoptosis and DNA fragmentation [67]. Moreover, the *H. dicksoniae* extract also contained caffeic acid (polyphenol), which is a secondary metabolite found in propolis, vegetables (potatoes and carrots), olives, fruits, and coffee beans. It mainly constitutes hydroxycinnamic acid in the human diet. Caffeic acid possesses high anticancer efficiency due to its proliferation-inhibiting, antioxidant, apoptosis-inducing, and anti-inflammatory features [68]. In addition, caffeic acid and its derivatives are known for their immunostimulatory, antibacterial, anti-atherosclerotic, antiviral, antidiabetic, antiproliferative, cardioprotective, anti-hepatocellular carcinoma, and hepatoprotective activities [69]. The other bioactive compounds that are not discussed in detail exhibited biological activities through their binding interaction with target proteins, showing that their activities are synergistic. Taken together, the GC-MS, LC-MS/MS, and molecular docking analyses revealed promising interactions between various bioactive compounds and target proteins associated with antimicrobial, antioxidant, and anticancer activities. The development of drugs and treatment schemes based on these compounds is becoming increasingly important, given the emerging resistance of numerous pathogens and the need to prevent and cure non-communicable diseases.

## 4. Materials and Methods

### 4.1. Plant Material

Plant samples (*H. dicksoniae* and *S. capensis*) were collected from the Al-Samman region in the northeastern part of the Kingdom of Saudi Arabia in February 2023. Both plants were identified at the Herbarium of the Desert Research Center, Egypt.

### 4.2. Plant Extract Preparation

The aerial parts of the plants (100 g of each) were shade-dried, ground, and separately soaked (24 h) in ethyl alcohol (ethanol) (70%, 500 mL) in conical flasks inside a water bath (45 °C). Whatman filter paper was used to filter the resulting extract, which was followed by the evaporation of the filtrates using a rotary evaporator. The extracts were stored at 4 °C for further analyses.

### 4.3. Antifungal and Antibacterial Activities

The agar well-diffusion method was employed to test the antimicrobial potential of the ethanolic extracts of *H. dicksoniae* and *S. capensis* [70]. Two standard strains of Gram-positive bacteria (*Bacillus subtilis* RCMB 015 (1) NRRL B-543 and *Staphylococcus aureus* ATCC 25923) and two Gram-negative bacteria (*Proteus vulgaris* RCMB 004 (1) ATCC 13315 and *Escherichia coli* ATCC 25922) were used for the antibacterial assay. Two clinically pathogenic fungi (*Aspergillus fumigatus* RCMB 002008 and *Candida albicans* RCMB 005003 (1) ATCC 10231) were used for the antifungal assay. A total of 100 μL of suspension containing 1 × 10^8^ colony-forming units (CFU)/mL of the tested bacteria and 1 × 10^4^ spore/mL of fungi was spread on nutrient agar and Sabouraud dextrose agar, respectively. Wells (6 mm in diameter) were made in the agar and loaded with 100 μL of the tested sample solutions in 1 mL DMSO with a concentration of 10 mg/mL. DMSO, employed to dissolve the tested samples, was used as a negative control, while ampicillin, gentamicin, and amphotericin B served as the positive controls against Gram-positive bacteria, Gram-negative bacteria, and fungi, respectively. The inoculated plates were incubated for 24 h at 37 °C for bacteria and 48 h at 28 °C for fungi. The diameter of the inhibition zone of growth was measured in millimeters (mm). The experiments were performed in triplicate, and the data were expressed as the mean ± standard deviation. The minimum inhibitory concentrations (MICs) of the plant extracts were determined via the microdilution method, and the MIC values were taken as the lowest sample concentration that prevented visible bacterial and fungal growth. The bacterial and fungal strains were obtained from the Culture Collections of the Regional Center of Mycology and Biotechnology (RCMB) at Al-Azhar University, Egypt.

### 4.4. Antioxidant Activity Assay

The antioxidant activity of the plant extracts was determined against 2,2-diphenyl-1-picrylhydrazyl (DPPH) according to the procedure of Gardeli et al. [71]. An ethanolic solution of DPPH radicals (0.004% *w*/*v*) was prepared and stored in the dark at 10 °C. Similarly, an ethanolic solution of the test compound was prepared. The ethanolic solution of *H. dicksoniae* extract (40 µL) at various concentrations (0–1000 μg/mL) was added to the DPPH solution (3 mL), and absorbance was immediately recorded using a UV–visible spectrophotometer (Milton Roy, Spectronic 1201, Houston, TX, USA). The decrease in the absorbance was continuously observed at 515 nm at an interval of 1 min until a stabilized absorbance (16 min) was achieved. Ascorbic acid was employed as the standard. Measurements were performed in triplicate, and means were calculated. The following formula was used to calculate the percentage inhibition (*PI*) of DPPH radicals:

(1) $$PI=[(\frac{AC-AT\;}{AC})\times 100]$$

where *AC* = absorbance of the control at t = 0 min, and *AT* = absorbance of the sample + DPPH at t = 16 min.

The 50% inhibitory concentration (IC_50_) was estimated from the graphic plots of the dose–response curve.

### 4.5. MTT Assay-Based Anticancer Analysis

HCT-116 cells (human colon cancer cell line) were used for cytotoxicity analysis with the ethanolic extract of *H. dicksoniae*. The HCT-116 cells were obtained from the American Type Culture Collection (ATCC, Rockville, MD) and grown on inactivated fetal calf serum (10%) and gentamicin (50 µg/mL)-supplemented RPMI-1640 medium. The cells were maintained at 37 °C under a humidified atmosphere with CO_2_ (5%) and were sub-cultured 2 to 3 times a week. Cytotoxicity was determined using the cell viability assay method [72,73]. Briefly, the tumor cells (5 × 10^4^ cell/well) were suspended in the medium in Corning^®^ 96-well tissue culture plates, followed by incubation for 24 h. Then, the tested compound (three replicates) was added to 96-well plates to obtain twelve concentrations. Six vehicle controls (media or 0.5% DMSO) were used for each 96-well plate. After incubation, an MTT assay was performed to calculate viable cells. Concisely, the media of the 96-well plates was replaced with fresh RPMI 1640 medium (100 µL) without phenol red, and 12 mM MTT stock solution (10 µL) (5 mg of MTT in 1 mL of PBS) was added to each well, including the untreated controls, followed by incubation for 4 h (37 °C and 5% CO_2_). Subsequently, an 85 µL aliquot was removed from the wells, and DMSO (50 µL) was added to each well, followed by thorough mixing with a pipette and incubation (37 °C, 10 min). Then, optical density was measured at 590 nm in a microplate reader (SunRise, TECAN, Inc., Temecula, CA, USA) to determine the viable cells; the viability percentage was calculated using the following formula: [(ODt/ODc)] × 100%. In this formula, ODt represents the mean optical density of treated wells, whereas ODc represents the mean optical density of untreated wells. The survival curve of each tumor cell line was obtained by plotting surviving cells against drug concentrations. GraphPad Prism software v9.2.0.332 (San Diego, CA, USA) was used to draw graphic plots of the dose–response curve for each concentration to estimate the 50% inhibitory concentration (IC_50_) [73,74].

### 4.6. GC-MS Analysis of Crude H. dicksoniae Extract

The ethanolic extract of *H. dicksoniae* was subjected to GC-MS analysis. The chemical composition of the samples was analyzed using a Trace GC1310-ISQ mass spectrometer (Thermo Scientific, Austin, TX, USA) equipped with a direct capillary column TG–5MS (30 m × 0.25 mm × 0.25 µm film thickness). The column oven temperature was initially held at 35 °C, then increased by 3 °C/min to 200 °C, and finally held for 3 min. Then, it was increased to a final temperature of 280 °C at a rate of 3 °C/min and held for 10 min. The temperatures of the injector and MS transfer line were kept at 250 and 260 °C, respectively. Helium was used as the carrier gas at a constant flow rate of 1 mL/min. The solvent delay time was 3 min, and 1 µL of diluted samples was injected automatically using the Autosampler AS1300 (Thermo Scientific, Austin, TX, USA) coupled with GC in the split mode. EI mass spectra were obtained at 70 eV ionization voltages over the range of *m*/*z* 40–1000 in full-scan mode. The ion source temperature was set at 200 °C. The components were identified by comparing their retention times and mass spectra with those of the WILEY 09 and NIST 11 mass-spectral database.

### 4.7. LC-MS/MS Analysis of Crude H. dicksoniae Extract

#### 4.7.1. Chemicals and Reagents

The methanol (Fisher Scientific, Loughborough, UK) and acetonitrile (Sigma-Aldrich, Hamburg, Germany) used in this study were of HPLC grade. Formic acid (≥98%) was purchased from Fisher Scientific (UK). MS-grade (≥98%) ammonium formate was purchased from Sigma Aldrich (Germany). Sodium hydroxide anhydrous pellets were purchased from Fisher Scientific (UK), and water was obtained from a Millipore (Milli-Q, Burlington, MA, USA, 18.2 MΩ·cm at 25 °C) water purification system.

#### 4.7.2. Sample Preparation

The lyophilized extract (50 mg) was dissolved in the solvent mixture (1 mL at a water/methanol/acetonitrile ratio of 50:25:25, *w*/*w*) to prepare the stock solution. The stock solution was vortexed (2 min) and subjected to ultra-sonication (10 min) followed by centrifugation (1000 rpm, 10 min). A sample aliquot (50 µL) was diluted to 1000 µL using the same solvent mixture (water/methanol/acetonitrile ratio of 50:25:25, *w*/*w*). The final injected concentration was 2.5 µg/µL.

#### 4.7.3. LC-MS/MS Analysis

Chromatographic separation was carried out in a Sciex ExionLC system (AB Sciex, Framingham, MA, USA) that was equipped with an autosampler system, an in-line filter disk pre-column (0.5 µm × 3.0 mm), and an X select HSS T3 C18 (100 A° 2.5 µm, 2.1 mm × 150 mm) column (Waters Corporation (NYSE:WAT), Milford, MA, USA). The column temperature was set at 40 °C. The mobile phase (A) comprised ammonium formate and (5 mM) methanol solution (1%) (pH = 3.0, adjusted with formic acid). The mobile phase (B) contained acetonitrile (100%) for the positive mode, whereas ammonium formate (5 mM) and methanol (1%) solution (pH = 8.0, adjusted with sodium hydroxide) were used in the negative mode as the mobile phase (C). The flow rate was adjusted to 0.3 mL/min and the injection volume was 10 µL. Table 8 summarizes the UPLC gradient.

The LC system was combined with a Sciex Triple TOF 5600 system that operated in electrospray ionization (ESI) mode to acquire spectra over a mass range of *m*/*z* 50–1000. The sprayer capillary and declustering potential voltages were 4500 and 80 eV in positive mode, respectively, and −4500 and −80 V in negative mode, respectively. The source temperature was set at 500 °C with the curtain gas at 25 psi, and GS1 and GS2 at 45 psi. The collision energy was set at 35 V in positive mode and −35 V in negative mode, with CES at 20 V (positive mode) and 15 V (negative mode). The ion tolerance was set at 10 ppm. TripleTOF5600+ was operated by following the information-dependent acquisition (IDA) protocol. MS and MS/MS data were collected using Analyst-TF 1.7.1 software.

#### 4.7.4. Data Processing

The MS-DIAL 4.9 (RIKEN) program was used for small-molecule non-targeting comprehensive analysis. The ReSpect negative (1573 records) and ReSpect positive (2737 records) databases were used for compound identification. The MS-DIAL 4.9 parameters for data collection were as follows: MS1 tolerance of 0.01 Da and MS2 tolerance of 0.05 Da. The minimum peak height was 1000 amplitudes, the mass slice width was 0.05 Da, the smoothing level was 2 scans, and the minimum peak width was 5 scans. An accurate mass tolerance in MS1 and MS2 of 0.2 Da and a score cut-off of 70% were set as the identification parameters.

### 4.8. Bioinformatics Experiments

#### 4.8.1. Ligand Preparation

Data on *H. dicksoniae* compounds were retrieved from the PubChem database. The MMFF94 force field was applied using Avogadro 1.2.0 software to perform ligand energy minimization [75], which served as the queries in the SwissTargetPrediction tool for target prediction [76,77].

#### 4.8.2. Protein Preparation

The target microbial proteins included *B. subtilis* penicillin-binding protein (dacC) (UniProt ID: P39844); *C. albicans* lanosterol 14-alpha demethylase (ERG11) (UniProt ID: P10613), C-22 sterol desaturase (ERG5) (UniProt ID: G1UB11), Delta(7)-sterol 5(6)-desaturase (ERG3) (UniProt ID: O93875), and C-8 sterol isomerase (ERG2) (UniProt ID: A0A1D8PCB9); *E. coli* 30S ribosomal protein S17 (rpsQ) (UniProt ID: P0AG63); *S. aureus* beta-lactam-inducible penicillin-binding protein (pbp) (UniProt ID: P07944); and *P. vulgaris* 30S ribosomal protein S1 (rpsA) (UniProt ID: A0A094TL12). For target prediction–repetition, three anticancer target proteins were selected, namely, carbonic anhydrase II CA2 (14 repeats) (UniProt ID: P00918); peroxisome proliferator-activated receptor alpha PPARA (9 repeats) (UniProt ID: Q07869); and protein kinase C epsilon PRKCE (4 repeats) (UniProt ID: Q02156). The three antioxidant target proteins were fatty acid-binding protein FABP2 (3 repeats) (UniProt ID: P12104); fatty acid-binding protein FABP3 (4 repeats) (UniProt ID: P05413); and fatty acid-binding protein FABP4 (3 repeats) (UniProt ID: P15090). The structures of the target proteins were retrieved from the UniprotKB database. DeepSite was used to predict the protein binding sites, except for those of *E. coli* and *P. vulgaris* [78]. AutoDock Tools (1.5.7) was employed for protein preparation, which involved the removal of water molecules, the addition of hydrogen atoms, and charge assignment [79].

#### 4.8.3. Molecular Docking

AutoDock Vina [80] and the HDock server [81] were used for molecular docking studies. The docking simulations were based on the ligand and protein structures, and the calculated free binding energy (ΔG) revealed the binding affinity. Finally, Biovia Discovery Studio [82] was used to visualize the docking results.

### 4.9. Statistical Analysis

The experiments were performed in triplicate, and the results were expressed as the mean ± standard error (SE). The results were statistically analyzed using one-way analysis of variance (ANOVA) and Tukey’s post hoc test; significantly different values were differentiated at *p* ≤ 0.05.

## 5. Conclusions

*H. dicksoniae* and *S. capensis* plant extracts were investigated for their antimicrobial (fungal and bacterial) efficacy. The ethanolic extract of *H. dicksoniae* presented a higher antimicrobial activity, and thus, it was further studied to evaluate its anticancer and antioxidant properties, which would favor its further application in future clinical trials. GC-MS and LC-MS/MS analyses combined with molecular docking studies revealed the binding interactions and mode of action of the identified bioactive compounds against various key target proteins, which confirmed their antimicrobial, antioxidant, and anticancer activities. Thus, the isolation of pure compounds from *H. dicksoniae* extract may play a significant role in the discovery and design of new drugs for the treatment of human diseases. Further studies will be conducted, focusing on the isolation of pure bioactive compounds using different organic solvents and evaluation of their activities against different pathogenic microorganisms and cancerous cell lines, as well as their free radical-scavenging ability, both in vitro and in vivo.

## Figures and Tables

**Table 1:** Antifungal and antibacterial activities of *H. dicksoniae* and *S. capensis* ethanolic extracts.

Tested Microorganisms		*H. dicksoniae*	*S. capensis*	Standard Treatment	
*Aspergillus fumigatus*	Fungi	NA	NA	17.0 ± 0.1	Amphotericin B
*Candida albicans*		20.6 ± 1.5	16.3 ± 0.6	21.9 ± 0.1	
*Staphylococcus aureus*	Gram (+) Bacteria	18.6 ± 0.6	17.8 ± 0.6	28.9 ± 0.1	Ampicillin
*Bacillus subtilis*		15.0 ± 0.6	13.0 ± 0.4	17.3 ± 0.1	
*Escherichia coli*	Gram (−) Bacteria	22.4 ± 0.6	21.0 ± 0.3	25.3 ± 0.2	Gentamicin
*Proteus vulgaris*		8.0 ± 0.6	NA	17.3 ± 0.1	

Antifungal and antibacterial activities are expressed in terms of inhibition diameter (mm). Values are presented as means ± SE (n = 3). NA: No activity.

**Table 2:** Minimum inhibitory concentrations (μg/mL) of *H. dicksoniae* and *S. capensis* extracts against tested microorganisms.

Tested Microorganisms		Minimum Inhibitory Concentration (MIC) (μg/mL)			
		*H. dicksoniae*	*S. capensis*	Standard Treatment	
*Candida albicans*	Fungi	1.95	31.25	0.98	Amphotericin B
*Staphylococcus aureus*	Gram (+) Bacteria	0.98	7.81	0.49	Ampicillin
*Bacillus subtilis*		7.81	12.81	0.49	
*Escherichia coli*	Gram (−) Bacteria	1.95	5.60	0.49	Gentamicin
*Proteus vulgaris*		3.90	NA	0.49	

NA: No activity.

**Table 3:** GC-MS analysis of *H. dicksoniae* ethanolic extract.

No.	Retention Time (min)	Compound	Area (%)	Formula	Molecular Weight
1	37.75	2(3H)-furanone, 5-heptyldihydro	0.70	C_11_H_20_O_2_	184
2	38.64	1,2-benzenedicarboxylic acid, diethyl ester (diethyl phthalate)	1.01	C_12_H_14_O_4_	222
3	42.04	1-(4-isopropylphenyl)-2-methylpropyl acetate	0.66	C_15_H_22_O_2_	234
4	45.57	Benzoic acid, phenylmethyl ester (benzyl benzoate)	14.31	C_14_H_12_O_2_	212
5	48.64	Isopropyl myristate	44.53	C_17_H_34_O_2_	270
6	51.51	Hexadecanoic acid, methyl ester	1.00	C_17_H_34_O_2_	270
7	53.04	n-Hexadecanoic acid	2.29	C_16_H_32_O_2_	256
8	53.70	Hexadecanoic acid, ethyl ester	0.22	C_18_H_36_O_2_	284
9	57.85	Octadecanoic acid, methyl ester	0.85	C_19_H_38_O_2_	298
10	60.35	Octadecanoic acid, ethyl ester	0.14	C_20_H_40_O_2_	312
11	63.33	Glycidyl palmitate	0.40	C_19_H_36_O_3_	312
12	71.52	Hexadecanoic acid, phenylmethyl ester	0.32	C_23_H_38_O_2_	346

**Table 4:** Binding affinity (∆G, kcal/mol)–based interaction strength between GC-MS-identified ligands and target proteins.

	Target Proteins	Anticancer			Antioxidant			Antimicrobial							
Ligands		CA2	PPARA	PRKCE	FABP2	FABP3	FABP4	*C. albicans*				*B. subtilis*	*S. aureus*	*P. vulgaris*	*E. coli*
								ERG2	ERG3	ERG5	ERG11	dacC	pbp	rpsA	rpsQ
Benzoic acid, phenylmethyl ester		**−6.0**	−6.8	**−7.1**	−8.2	**−7.0**	−6.8	**−8.3**	−7.0	**−7.9**	**−7.2**	**−6.1**	**−6.6**	−5.0	**−4.9**
1-(4-isopropylphenyl)-2-methylpropyl acetate		−5.2	−6.4	−6.8	−7.5	−6.5	**−6.9**	−8.0	**−7.3**	−6.5	−6.7	−6.0	−6.5	**−5.3**	−4.5
Hexadecanoic acid, phenylmethyl ester		−5.3	**−7.4**	−5.9	**−8.5**	−6.5	−6.4	−7.8	−7.1	−6.7	−6.4	−5.3	−5.4	−4.2	−3.3

Bold values indicate the highest binding affinity (i.e., the most negative ∆G values), reflecting strong interactions between the ligand and the active site of the target protein.

**Table 5:** Molecular docking analysis between the GC-MS-identified ligands and a representative receptor from each set of target proteins revealed the compounds with the highest binding affinity (i.e., the most negative ∆G values).

Dock Score (kcal/mol) of Ligand–Protein Interaction *	2D Binding Interaction	3D Binding Interaction
Hexadecanoic acid, phenylmethyl ester with PPARA (∆G = −7.4 kcal/mol)	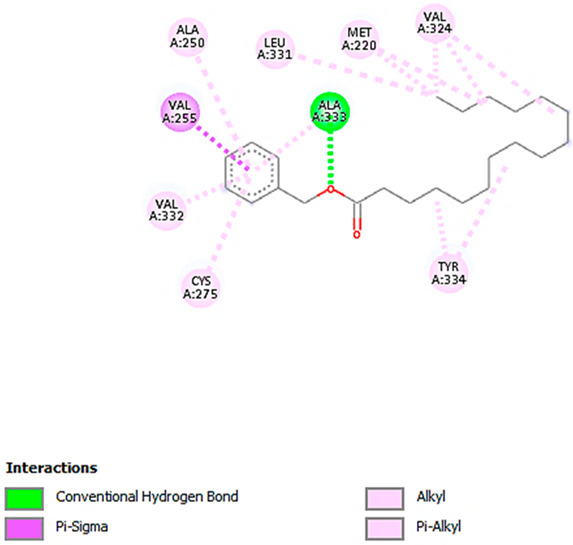	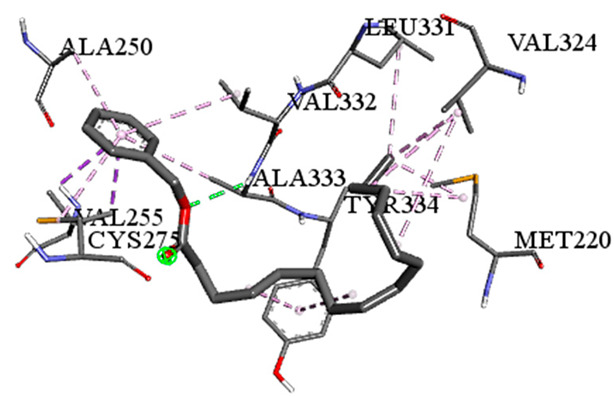
Hexadecanoic acid, phenylmethyl ester with FABP2 (∆G = −8.5 kcal/mol)	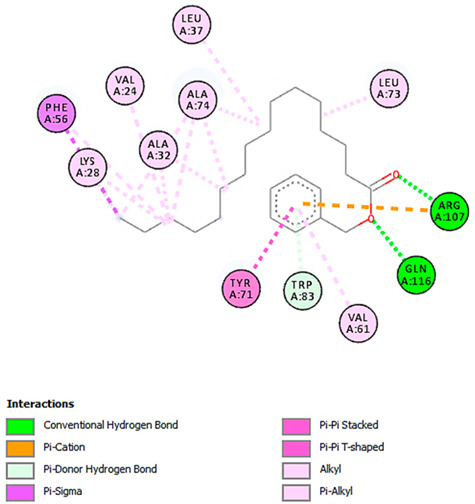	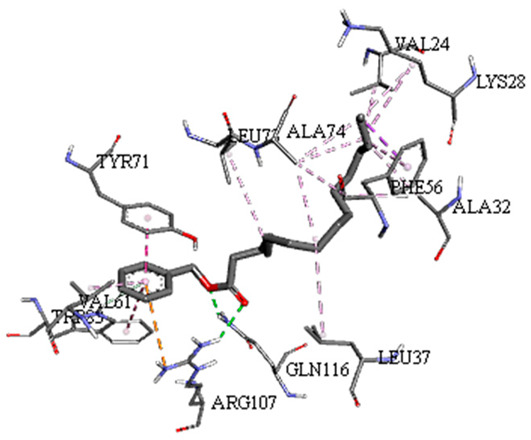
Benzoic acid, phenylmethyl ester with ERG2-*C. albicans* (∆G = −8.3 kcal/mol)	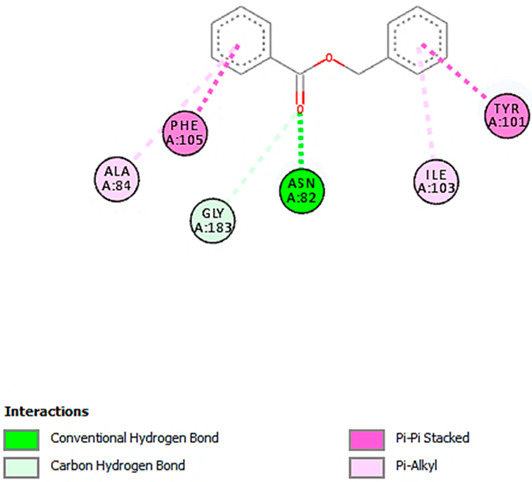	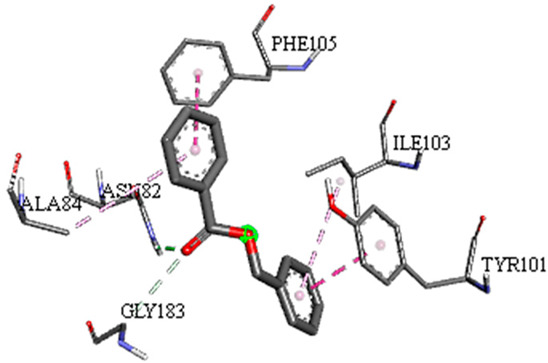
Benzoic acid, phenylmethyl ester with pbp-*S. aureus* (∆G = −6.6 kcal/mol)	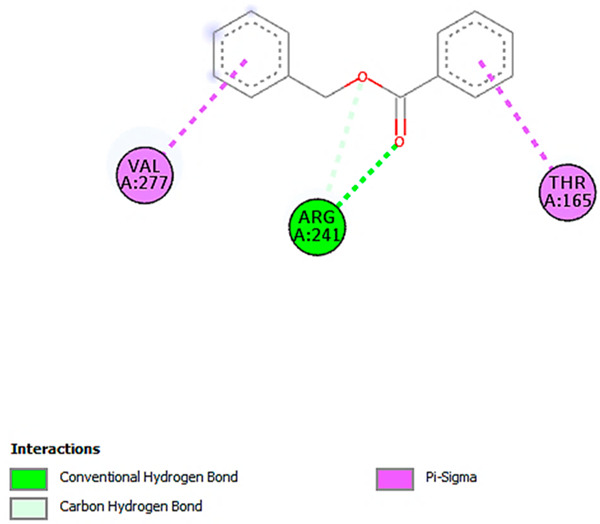	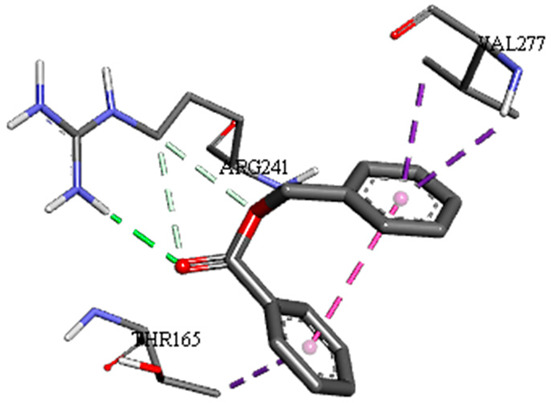
1-(4-isopropylphenyl)-2-methylpropyl acetate with rpsA-*P. vulgaris* (∆G = −5.3 kcal/mol)	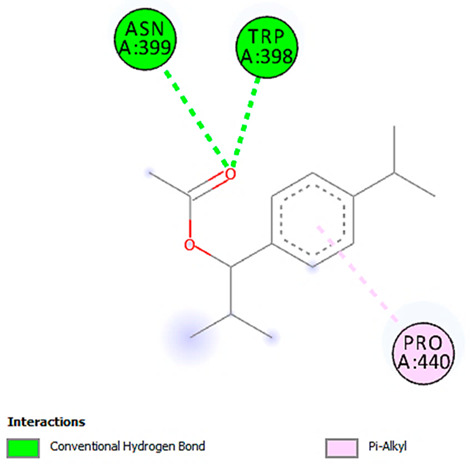	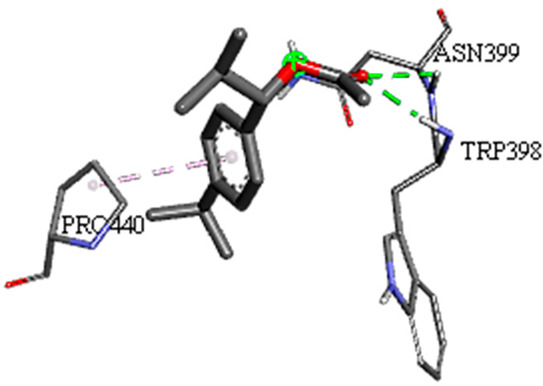

* Only the binding interactions between the ligand and one representative receptor from each set of target proteins exhibiting the highest binding affinity (i.e., the most negative ∆G values) are shown here. See Table S3 in the Supplementary Materials for all interactions.

**Table 6:** Binding affinity (∆G, kcal/mol)–based interaction strength between LC-MS/MS-identified ligands and target proteins.

	Target Proteins	Anticancer			Antioxidant			Antimicrobial							
Ligands		CA2	PPARA	PRKCE	FABP2	FABP3	FABP4	*C. albicans*				*B. subtilis*	*S. aureus*	*P. vulgaris*	*E. coli*
								ERG2	ERG3	ERG5	ERG11	dacC	pbp	rpsA	rpsQ
Negative ion mode compounds (MS)															
Acacetin		−6.3	−7.0	−8.0	−8.2	−7.8	−8.1	**−9.2**	**−8.7**	−8.1	−7.9	−7.9	−8.1	−6.5	−5.9
Hesperetin		−6.5	−7.5	−8.6	−8.5	−8.3	−8.3	−8.9	**−8.7**	−7.7	−7.8	−7.8	−8.3	−6.7	−6.0
Kaempferol-3-glucuronide		−6.8	−8.3	−8.4	**−9.3**	−9.2	−9.5	−7.0	−6.5	−8.8	−8.7	−8.7	−7.8	−6.6	−5.6
Kaempferol-7-neohesperidoside		**−8.0**	−7.8	**−9.8**	−9.1	−6.5	−9.4	−8.0	−6.6	−9.4	−9.1	−9.1	−9.0	−7.3	−6.4
Positive ion mode compounds (MS)															
3,5,7-trihydroxy-4′-methoxyflavone		−6.6	−7.3	−8.4	−8.3	−7.9	−8.2	−9.1	**−8.7**	−8.4	−7.7	−7.6	−8.0	−6.7	−5.8
Apigenin 8-C-glucoside		−6.6	−7.3	−8.7	−9.1	**−9.9**	−9.5	−8.0	−6.6	−8.5	−8.5	−8.0	−8.1	−6.5	−6.2
Cyanidin-3-O-rutinoside		−7.0	−8.6	−8.5	−7.1	−9.1	**−10.4**	−7.1	−6.5	−9.2	−9.1	**−9.2**	−8.7	−6.9	**−7.2**
Luteolin-3′,7-di-O-glucoside		−7.2	**−9.2**	−8.9	−7.0	−6.9	−9.2	−7.3	−6.9	**−9.7**	**−9.6**	−8.7	**−9.6**	**−7.9**	−6.6

Bold values indicate the highest binding affinity (i.e., the most negative ∆G values), reflecting strong interactions between the ligand and the active site of the target protein.

**Table 7:** Molecular docking analysis between the LC-MS/MS-identified ligands and a representative receptor from each set of target proteins revealed the compounds with the highest binding affinity (i.e., the most negative ∆G values).

Dock Score (kcal/mol) of Ligand–Protein Interaction *	2D Binding Interaction	3D Binding Interaction
Kaempferol-7-neohesperidoside with PRKCE (∆G = −9.8 kcal/mol)	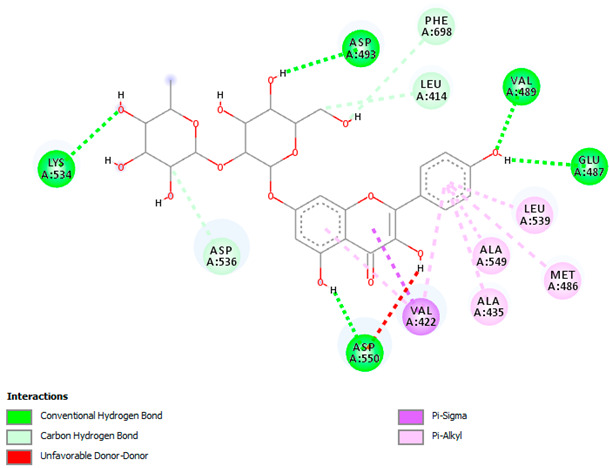	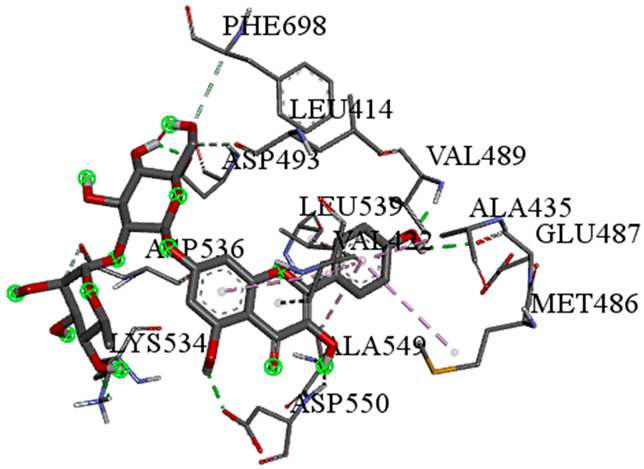
Cyanidin-3-O-rutinoside with FABP4 (∆G = −10.4 kcal/mol)	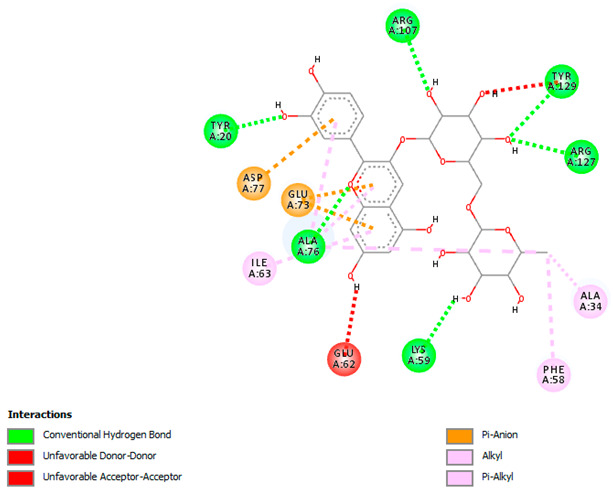	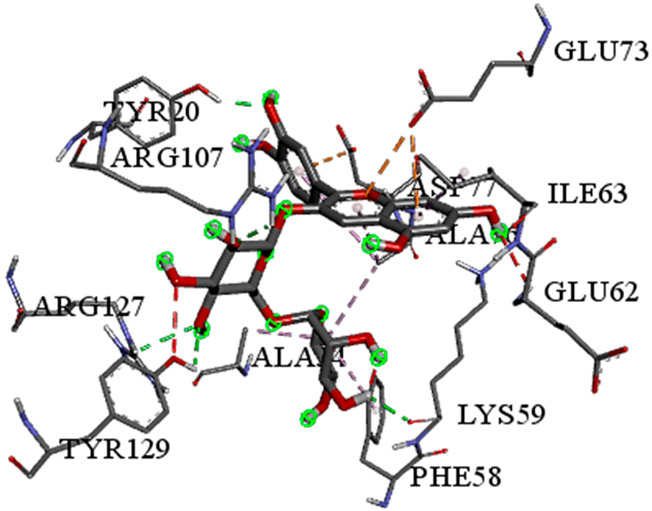
Luteolin-3′,7-di-O-glucoside with ERG5-*C. albicans* (∆G = −9.7 kcal/mol)	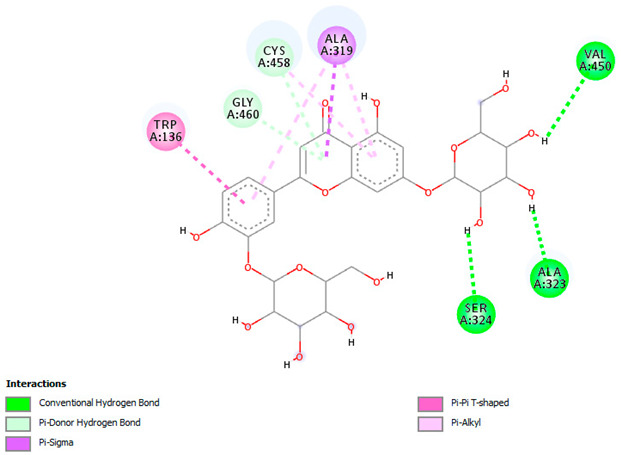	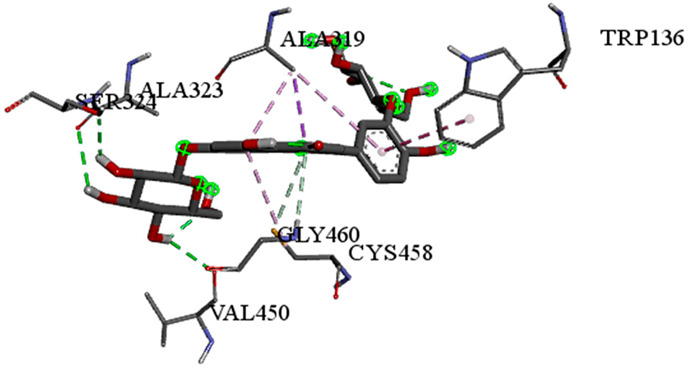
Luteolin-3′,7-di-O-glucoside with pbp-*S. aureus* (∆G = −9.6 kcal/mol)	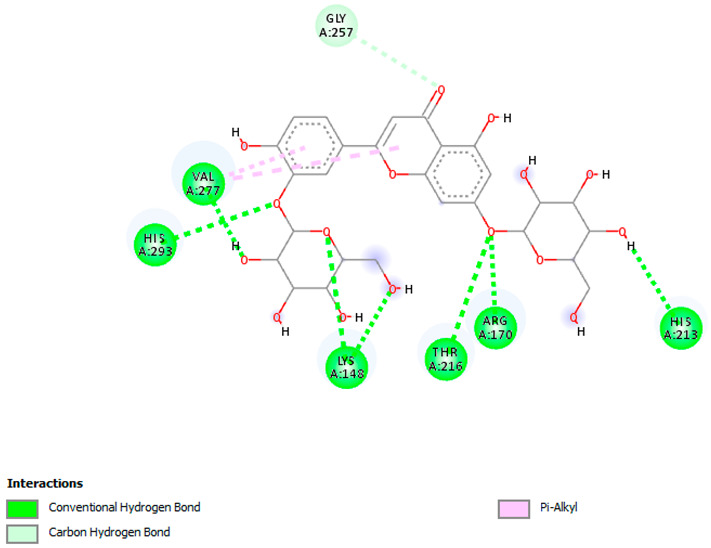	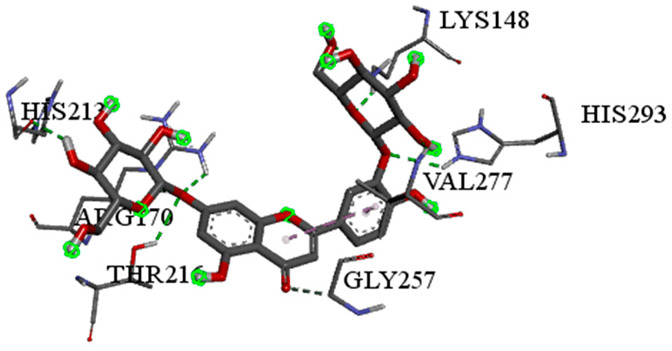
Luteolin-3′,7-di-O-glucoside with rpsA-*P. vulgaris* (∆G = −7.9 kcal/mol)	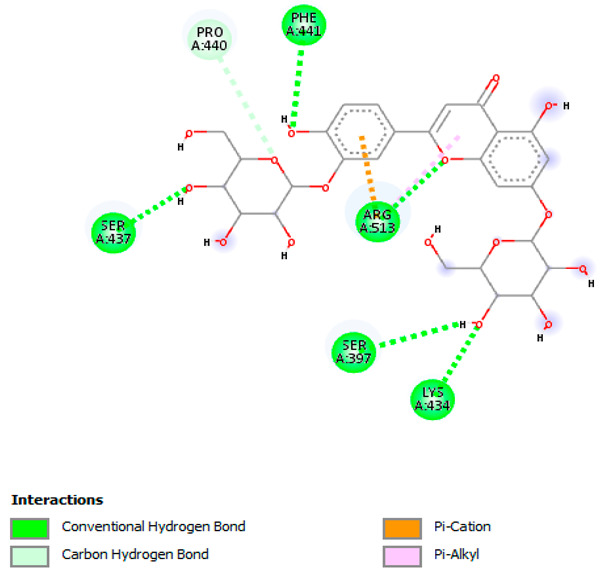	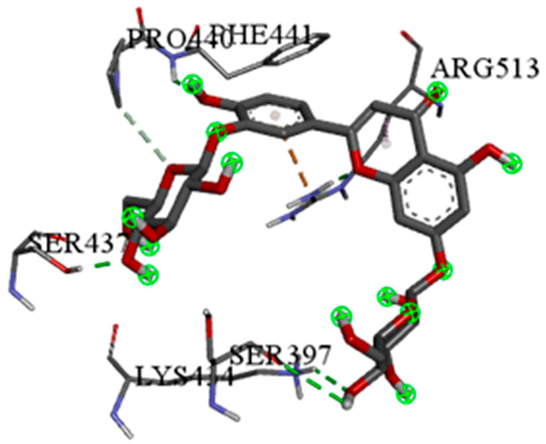

* Only the binding interactions between the ligand and one representative receptor from each set of target proteins exhibiting the highest binding affinity (i.e., the most negative ∆G values) are shown here. See Table S5 in the Supplementary Materials for all interactions.

**Table 8:** UPLC gradient elution program.

Time (min)	Mobile Phase A (or B)	Mobile Phase C
0	95	5
1	95	5
21	5	95
28	5	95
28.1	95	5
35	95	5

## Data Availability

The data that support the findings of this study are incorporated into the article.

## Supplementary Materials

The following supporting information can be downloaded at: https://www.mdpi.com/article/10.3390/ph18050765/s1, Figure S1. Mass spectra and chemical structures of GC-MS-identified compounds in the ethanolic extract of *H. dicksoniae*; Table S1. LC-MS/MS analysis of the ethanolic extract of *H. dicksoniae* in both negative and positive ionization modes; Table S2. Binding affinity (∆G, kcal/mol)-based interaction strength between all GC-MS-identified ligands and target proteins; Table S3. Molecular docking analysis showing 2D and 3D binding interactions between GC-MS-identified ligands and target proteins with the highest binding affinity (i.e., the most negative ∆G values); Table S4. Binding affinity (∆G, kcal/mol)-based interaction strength between all the LC-MS/MS–identified ligands and target proteins; Table S5. Molecular docking analysis showing 2D and 3D binding interactions between LC-MS/MS-identified ligands and target proteins with the highest binding affinity (i.e., the most negative ∆G values).

## References

[1] Fouda H., Abdulkader O., Sharaf A.E., Elhaw M. (2022). Phytochemical constituents and gas chromatography with mass spectroscopy analysis of *Euphorbia heterophylla*’s aerial parts. Al-Azhar J. Pharm. Sci..

[2] El-Sohaimy S., Hamad G., Mohamed S., Amar M., Al-Hindi R. (2015). Biochemical and functional properties of *Moringa oleifera* leaves and their potential as a functional food. Glob. Adv. Res. J. Agric. Sci..

[3] Izuegbuna O. (2022). Leukemia chemoprevention and therapeutic potentials: Selected medicinal plants with anti-leukemic activities. Nutr. Cancer.

[4] Muema F.W., Nanjala C., Oulo M.A., Wangchuk P. (2023). Phytochemical content and antidiabetic properties of most commonly used antidiabetic medicinal plants of Kenya. Molecules.

[5] Ali S.I., Sheikh W.M., Rather M.A., Venkatesalu V., Muzamil Bashir S., Nabi S.U. (2021). Medicinal plants: Treasure for antiviral drug discovery. Phytother. Res..

[6] Qari S.H., Abdulmajeed F., Al Refaei W.F., Alaa Q. (2021). Exploration of the Medicinal Flora of the Aljumum Region in Saudi Arabia. Appl. Sci..

[7] Abdelwahab M.F., Sangi S., Arafat H.H., Ragab E.A. (2016). New phytochemical constituent and bioactivities of *Horwoodia dicksoniae* and *Rumex cyprius*. Pharmacogn. Mag..

[8] Francis A., Lujan-Toro B.E., Warwick S.I., Macklin J.A., Martin S.L. (2021). Update on the Brassicaceae species checklist. Biodivers. Data. J..

[9] Banan S.A., Al-Watban A.A., Doaigey A.R., Alsahli A.A. (2019). Anatomical adaptations in species of Poaceae growing in Al-Ha’ir region of Riyadh, Saudi Arabia. Afr. J. Plant. Sci..

[10] Khalil A.M.A., Saleh A.M., Abo-El-Souad S.M.S., Mohamed M.S.M. (2023). Plants from a semi-arid environment as a source of phytochemicals against *Fusarium* crown and foot rot in zucchini. AMB. Expr..

[11] Fawzy G.A., Al-Taweel A.M., Abdel Baky N.A., Marzouk M.S. (2012). Cytotoxic and renoprotective flavonoid glycosides from *Horwoodia dicksoniae*. AJPP.

[12] Imran M., Rauf A., Abu-Izneid T., Nadeem M., Shariati M.A., Khan I.A., Imran A., Orhan I.E., Rizwan M., Atif M. (2019). Luteolin, a flavonoid, as an anticancer agent: A review. Biomed. Pharmacother..

[13] Singh A., Yadav S., Pathak P., Verma A., Yadav J.P. (2024). Harnessing Luteolin’s therapeutic potential in human disorders: Medicinal significance, biological, clinical properties and analytical aspects. Pharmacol. Res. Mod. Chin. Med..

[14] Yoo H.S., Won S.B., Kwon Y.H. (2022). Luteolin induces apoptosis and autophagy in HCT116 colon cancer cells via p53-dependent pathway. Nutr. Cancer.

[15] Ballash G.A., Parker E.M., Mollenkopf D.F., Wittum T.E. (2024). The One Health dissemination of antimicrobial resistance occurs in both natural and clinical environments. J. Am. Vet. Med. Assoc..

[16] Bertagnolio S., Dobreva Z., Centner C.M., Olaru I.D., Donà D., Burzo S., Huttner B.D., Chaillon A., Gebreselassie N., Wi T. (2024). WHO global research priorities for antimicrobial resistance in human health. Lancet Microbe.

[17] Nasim N., Sandeep I.S., Mohanty S. (2022). Plant-derived natural products for drug discovery: Current approaches and prospects. Nucleus.

[18] Zouine N., El Ghachtouli N., El Abed S., Ibnsouda Koraichi S. (2024). A comprehensive review on medicinal plant extracts as antibacterial agents: Factors, mechanism insights and future prospects. Sci. Afr..

[19] Cowan M.M. (1999). Plant products as antimicrobial agents. Clin. Microbiol. Rev..

[20] Saini N., Gahlawat S.K., Lather V., Gahlawat S., Salar R., Siwach P., Duhan J., Kumar S., Kaur P. (2017). Flavonoids: A nutraceutical and its role as anti-inflammatory and anticancer agent. Plant Biotechnology: Recent Advancements and Developments.

[21] Singh S., Gupta P., Meena A., Luqman S. (2020). Acacetin, a flavone with diverse therapeutic potential in cancer, inflammation, infections and other metabolic disorders. Food Chem. Toxicol..

[22] Dbeibia A., Taheur F.B., Altammar K.A., Haddaji N., Mahdhi A., Amri Z., Mzoughi R., Jabeur C. (2022). Control of *Staphylococcus aureus* methicillin resistant isolated from auricular infections using aqueous and methanolic extracts of *Ephedra alata*. Saudi J. Biol. Sci..

[23] Panche A.N., Diwan A.D., Chandra S.R. (2016). Flavonoids: An overview. J. Nutr. Sci..

[24] Van Heijenoort J. (2001). Formation of the glycan chains in the synthesis of bacterial peptidoglycan. Glycobiology.

[25] Farkaš V. (2003). Structure and biosynthesis of fungal cell walls Methodological approaches. Folia. Microbiol..

[26] Abu-Shanab B., Adwan G., Abu Safiya D., Adwan K., Abu-Shanab M. (2005). Antibacterial activity of *Rhus coriaria* extracts growing in Palestine. IUG Nat. Sci. Ser..

[27] Darah I., Lim S.H., Ninthianantham K. (2013). Effects of methanolic extract of *Wedelia chinensis* Osbeck (Asteraceae) leaves against pathogenic bacteria with emphasize on *Bacillus cereus*. Ind. J. Pharm. Sci..

[28] Sahgal G., Ramanathan S., Sasidharan S., Mordi M.N., Ismail S., Mansor S.M. (2011). In vitro and in vivo anticandidal activity of *Swietenia mahogany* methanolic seed extract. Trop. Biomed..

[29] Sohaib M., Al-Barakah F.N., Migdadi H.M., Husain F.M. (2022). Comparative study among *Avicennia marina*, *Phragmites australis*, and *Moringa oleifera* based ethanolic-extracts for their antimicrobial, antioxidant, and cytotoxic activities. Saudi J. Biol. Sci..

[30] Dzotam J.K., Touani F.K., Kuete V. (2016). Antibacterial and antibiotic—Modifying activities of three food plants (*Xanthosomamafaffa.* Lam., *Moringa oleifera* (L.). Schott and *Passiflora eduilis* Sims) against multidrug resistant (MDR) Gram–negative bacteria. BMC Complement. Altern. Med..

[31] Bukar A.M., Kyari M.Z., Gwaski P.A., Gudusu M., Kuburi F.S., Abadam Y.I. (2015). Evaluation of phytochemical and potential antibacterial activity of *Ziziphus spina-christi* against some medically important pathogenic bacteria. J. Pharmacogen. Phytochem..

[32] Shimada T. (2006). Salivary proteins as a defense against dietary tannins. J. Chem. Ecol..

[33] El Bouchti M., Bourhia M., Alotaibi A., Aghmih K., Majid S., Ullah R., Salamatullah A.M., El Achaby M., Oumam M., Hannache H. (2021). *Stipa tenacissima* L.: A new promising source of bioactive compounds with antioxidant and anticancer potentials. Life.

[34] Akhtar M.S., Hossain M.A., Said S.A. (2017). Isolation and characterization of antimicrobial compound from the stem-bark of the traditionally used medicinal plant *Adenium obesum*. J. Tradit. Complement. Med..

[35] Mehmood F., Hassan F., Sarfraz R., Khadim Z., Alamer K.H., Attia H., Saleh M.A., Al-Robai S.A., Zaman Q.U., Iftikhar Z. (2024). Phytochemical screening, antibacterial, antioxidant, and cytotoxic activities of *Geranium pusillum* leaves. Microsc. Res. Tech..

[36] Wu S., Tian L. (2017). Diverse phytochemicals and Bioactivities in the ancient fruit and modern functional food pomegranate. Molecules.

[37] Kumar G.P., Singh S.B. (2011). Antibacterial and antioxidant activities of ethanol extracts from trans Himalayan medicinal plants. EJAS.

[38] Sun M.Y., Bhaskar S.M.M. (2022). When two maladies meet: Disease burden and pathophysiology of stroke in cancer. Int. J. Mol. Sci..

[39] Yuan M., Zhang G., Bai W., Han X., Li C., Bian S. (2022). The role of bioactive compounds in natural products extracted from plants in cancer treatment and their mechanisms related to anticancer effects. Oxidative Med. Cell. Longev..

[40] Khan M., Khan M., Adil S.F., Alkhathlan H.Z. (2021). Screening of potential cytotoxic activities of some medicinal plants of Saudi Arabia. Saudi J. Biol. Sci..

[41] Khan S.U., Ullah F., Mehmood S., Fahad S., Ahmad R.A., Althobaiti F., Dessoky E.S., Saud S., Danish S., Datta R. (2022). Antimicrobial, antioxidant and cytotoxic properties of *Chenopodium glaucum* L.. PLoS ONE.

[42] Lazzeri L., Malaguti L., Bagatta M., D’Avino L. (2013). Characterization of the main glucosinolate content and fatty acid composition in no food Brassicaceae seeds. Acta Hortic..

[43] Das U.N. (2000). Beneficial effect(s) of n-3 fatty acids in cardiovascular diseases: But, why and how?. Prostaglandins Leukot. Essent. Fatty Acids.

[44] Grundy S.M. (1997). What is the desirable ratio of saturated, polyunsaturated, and monounsaturated fatty acids in the diet?. Am. J. Clin. Nutr..

[45] Casillas-Vargas G., Ocasio-Malavé C., Medina S., Morales-Guzmán C., Del Valle R.G., Carballeira N.M., Sanabria-Ríos D.J. (2021). Antibacterial fatty acids: An update of possible mechanisms of action and implications in the development of the next-generation of antibacterial agents. Prog. Lipid Res..

[46] Sehim A.E., Amin B.H., Yosri M., Salama H.M., Alkhalifah D.H., Alwaili M.A., Abd Elghaffar R.Y. (2023). GC-MS analysis, antibacterial, and anticancer activities of *Hibiscus sabdariffa* L. methanolic extract: In vitro and in silico studies. Microorganisms.

[47] Shaaban M.T., Ghaly M.F., Fahmi S.M. (2021). Antibacterial activities of hexadecanoic acid methyl ester and green–synthesized silver nanoparticles against multidrug–resistant bacteria. J. Basic Microbiol..

[48] Pakki E., Rewa M., Irma N. (2020). The Effectiveness of Isopropyl Myristate as Enhancing Agent in the Antioxidant Cream of Kasumba Turate Seed (*Carthamus tinctorius* L.). J. Pharm. Med. Sci..

[49] Ringertz S., Ringertz O. (1982). Antimicrobial effect of isopropil myristate when used as solvent in sterility testing. Pharm. Acta Helv..

[50] Huang L., Zhu X., Zhou S., Cheng Z., Shi K., Zhang C., Shao H. (2021). Phthalic acid esters: Natural sources and biological activities. Toxins.

[51] Aparna V., Dileep K.V., Mandal P.K., Karthe P., Sadasivan C., Haridas M. (2012). Anti-inflammatory property of n-hexadecanoic acid: Structural evidence and kinetic assessment. Chem. Biol. Drug Des..

[52] Farooqi S.S., Naveed S., Qamar F., Sana A., Farooqi S.H., Sabir N., Mansoor A., Sadia H. (2024). Phytochemical analysis, GC-MS characterization and antioxidant activity of *Hordeum vulgare* seed extracts. Heliyon.

[53] Lim J.H., Gerhart-Hines Z., Dominy J.E., Lee Y., Kim S., Tabata M., Xiang Y.K., Puigserver P. (2013). Oleic acid stimulates complete oxidation of fatty acids through protein kinase A-dependent activation of SIRT1- PGC1α complex. J. Biol. Chem..

[54] Wilsy J.I., Beschi D.A., Appavoo M.R., Wilsy J.I. (2021). GC-MS analysis, collected from Kavalkinaru area, Tirunelveli District, Tamil Nadu, India. Eur. J. Mol. Clin..

[55] Zai-Chang Y., Bo-Chu W., Xiao-Sheng Y., Qiang W. (2005). Chemical composition of the volatile oil from *Cynanchum stauntonii* and its activities of anti-influenza virus. Colloids Surf. B Biointerfaces.

[56] Haq N., Muhammad A.S., Saima M. (2018). Phytochemical composition and antioxidant potential of brassica. Brassica Germplasm. Charact. Breed. Util..

[57] Mattosinhos P.D.S., Sarandy M.M., Novaes R.D., Esposito D., Gonçalves R.V. (2022). Anti-inflammatory, antioxidant, and skin regenerative potential of secondary metabolites from plants of the Brassicaceae family: A systematic review of in vitro and in vivo preclinical evidence (Biological activities brassicaceae skin diseases). Antioxidants.

[58] Ecevit K., Barros A.A., Silva J.M., Reis R.L. (2022). Preventing microbial infections with natural phenolic compounds. Future Pharmacol..

[59] Patra A.K. (2012). An overview of antimicrobial properties of different classes of phytochemicals. Dietary Phytochemicals and Microbes.

[60] Dias M.C., Pinto D.C.G.A., Silva A.M.S. (2021). Plant flavonoids: Chemical characteristics and biological activity. Molecules.

[61] Yu X., Liu Z., Yu Y., Qian C., Lin Y., Jin S., Wu L., Li S. (2024). Hesperetin promotes diabetic wound healing by inhibiting ferroptosis through the activation of SIRT3. Phytother Res..

[62] De Lellis L., Cimini A., Veschi S., Benedetti E., Amoroso R., Cama A., Ammazzalorso A. (2018). The anticancer potential of peroxisome proliferator-activated receptor antagonists. Chem. Med. Chem..

[63] Chagas M.D.S.S., Behrens M.D., Moragas-Tellis C.J., Penedo G.X.M., Silva A.R., Gonçalves-de-Albuquerque C.F. (2022). Flavonols and flavones as potential anti-inflammatory, antioxidant, and antibacterial compounds. Oxid. Med Cell. Longev..

[64] Salehi B., Sharifi-Rad J., Cappellini F., Reiner Ž., Zorzan D., Imran M., Sener B., Kilic M., El-Shazly M., Fahmy N.M. (2020). The therapeutic potential of anthocyanins: Current approaches based on their molecular mechanism of action. Front. Pharmacol..

[65] Guaita-Esteruelas S., Gumà J., Masana L., Borràs J. (2018). The peritumoural adipose tissue microenvironment and cancer. The roles of fatty acid binding protein 4 and fatty acid binding protein 5. Mol. Cell. Endocrinol..

[66] Qattan M.Y., Khan M.I., Alharbi S.H., Verma A.K., Al-Saeed F.A., Abduallah A.M., Al Areefy A.A. (2022). Therapeutic importance of kaempferol in the treatment of cancer through the modulation of cell signalling pathways. Molecules.

[67] Periferakis A., Periferakis K., Badarau I.A., Petran E.M., Popa D.C., Caruntu A., Costache R.S., Scheau C., Caruntu C., Costache D.O. (2022). Kaempferol: Antimicrobial properties, sources, clinical, and traditional applications. Int. J. Mol. Sci..

[68] Ceylan F.D., Günal-Köroğlu D., Saricaoglu B., Ozkan G., Capanoglu E., Calina D., Sharifi-Rad J. (2024). Anticancer potential of hydroxycinnamic acids: Mechanisms, bioavailability, and therapeutic applications. Naunyn-Schmiedeberg’s Arch. Pharmacol..

[69] Espíndola K.M.M., Ferreira R.G., Narvaez L.E.M., Silva-Rosario A.C.R., Da-Silva A.H.M., Silva A.G.B., Vieira A.P.O., Monteiro M.C. (2019). Chemical and pharmacological aspects of caffeic acid and its activity in hepatocarcinoma. Front. Oncol..

[70] Scott A.C. (1989). Laboratory control of antimicrobial therapy. Mackie & McCartney Practical Medical Microbiology.

[71] Gardeli C., Papageorgiou V., Mallouchos A., Kibouris T., Komaitis M. (2008). Essential oil composition of *Pistacia lentiscus* L. and *Myrtus communis* L.: Evaluation of antioxidant capacity of methanolic extracts. Food Chem..

[72] Gomha S.M., Riyadh S.M., Mahmmoud E.A., Elaasser M.M. (2015). Synthesis and anticancer activities of thiazoles, 1,3-thiazines, and thiazolidine using chitosan-Grafted-Poly (vinylpyridine) as basic catalyst. Heterocycles.

[73] Mosmann T. (1983). Rapid colorimetric assay for cellular growth and survival: Application to proliferation and cytotoxicity assays. J. Immunol. Methods.

[74] Elaasser M.M., Abdel-Aziz M.M., El-Kassas R.A. (2011). Antioxidant, antimicrobial, antiviral and antitumor activities of pyranone derivative obtained from *Aspergillus candidus*. J. Microbiol. Biotech. Res..

[75] Hanwell M.D., Curtis D.E., Lonie D.C., Vandermeersch T., Zurek E., Hutchison G.R. (2012). Avogadro: An advanced semantic chemical editor, visualization, and analysis platform. J. Cheminf..

[76] Daina A., Michielin O., Zoete V. (2019). SwissTargetPrediction: Updated data and new features for efficient prediction of protein targets of small molecules. Nucleic Acids Res..

[77] Pires D.E., Blundell T.L., Ascher D.B. (2015). Platinum: A database of computationally modelled mammalian transcription factor DNA-binding preferences. Nucleic Acids Res..

[78] Jiménez J., Doerr S., Martínez-Rosell G., Rose A.S., De-Fabritiis G. (2017). DeepSite: Protein-binding site predictor using 3D-convolutional neural networks. Bioinformatics.

[79] Morris G.M., Huey R., Lindstrom W., Sanner M.F., Belew R.K., Goodsell D.S., Olson A.J. (2009). AutoDock4 and AutoDockTools4: Automated docking with selective receptor flexibility. J. Comput. Chem..

[80] Trott O., Olson A.J. (2010). AutoDock Vina: Improving the speed and accuracy of docking with a new scoring function, efficient optimization, and multithreading. J. Comput. Chem..

[81] Cabalu J., Malabanan M.M., Villarante N.R., Este A.B., Filomon M.M., Gutierrez J.A. (2020). HDock: A Hadoop-based docking algorithm to tackle molecular docking at large scale. J. Mol. Model..

[82] Dassault Systèmes BIOVIA (2020). Discovery Studio Visualizer.

