# Supplementary material for: Unveiling Therapeutic Powers of Indigenous Flora: Antimicrobial, Antioxidant, and Anticancer Properties of Horwoodia dicksoniae
Source: Pharmaceuticals (Basel). 2025 May 21;18(5):765. doi: 10.3390/ph18050765 (PMC12114875; doi:10.3390/ph18050765)
Supplement: Supplementary file 1 [file pharmaceuticals-18-00765-s001.zip › pharmaceuticals-3118368-supplementary.pdf]

Supplementary File

Figure S1. Mass spectra and chemical structures of GC-MS-identified compounds in the ethanolic extract of *H. dicksoniae*.

1

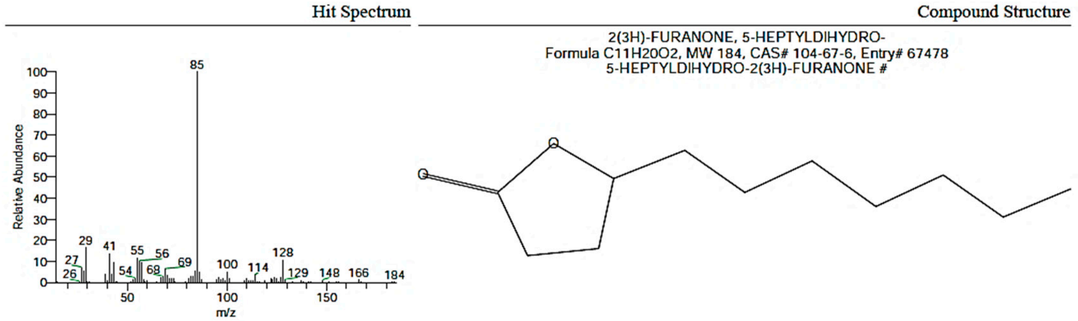

2

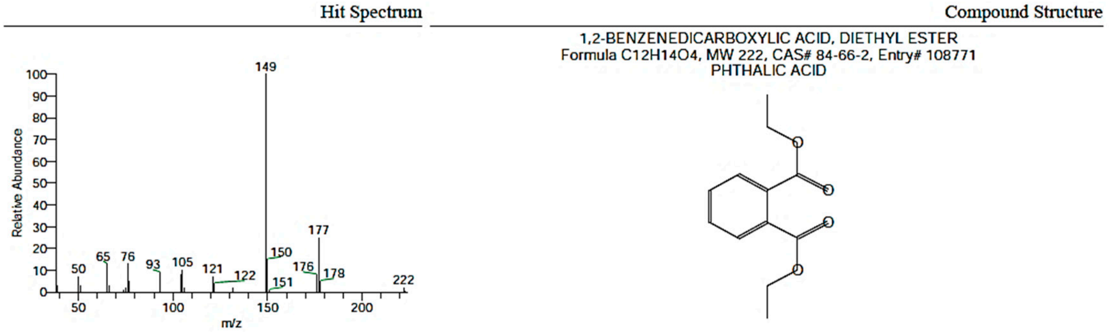

3

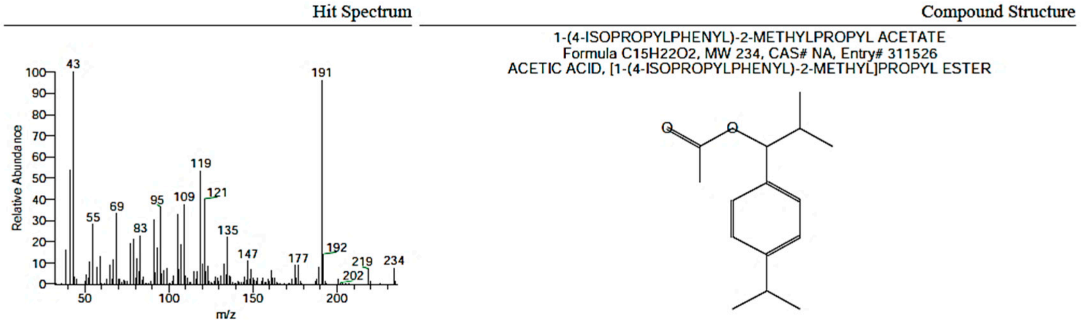

4

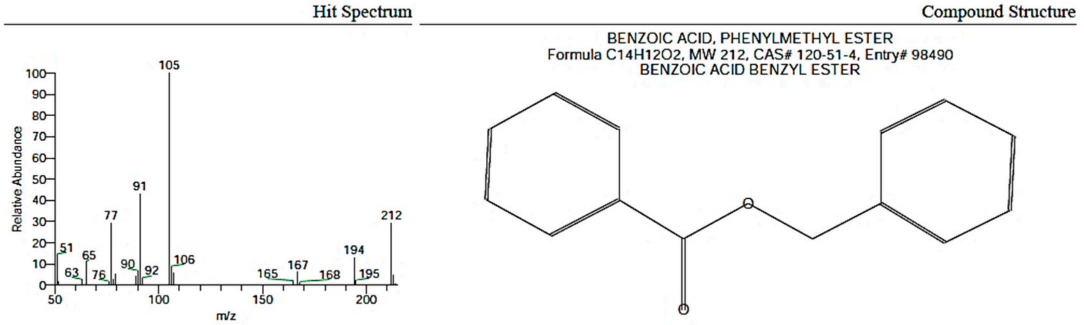

5	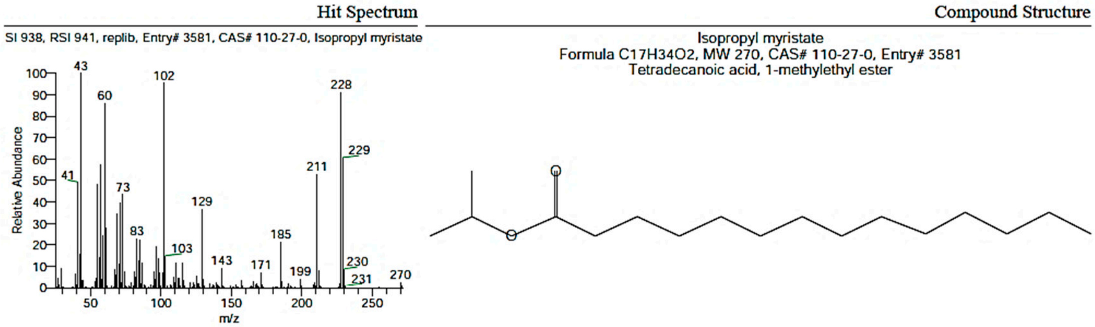Hit Spectrum<br>Compound Structure<br>SI 938, RSI 941, replib, Entry# 3581, CAS# 110-27-0, Isopropyl myristate<br>Isopropyl myristate<br>Formula C17H34O2, MW 270, CAS# 110-27-0, Entry# 3581<br>Tetradecanoic acid, 1-methylethyl ester<br>Relative Abundance<br>m/z
6	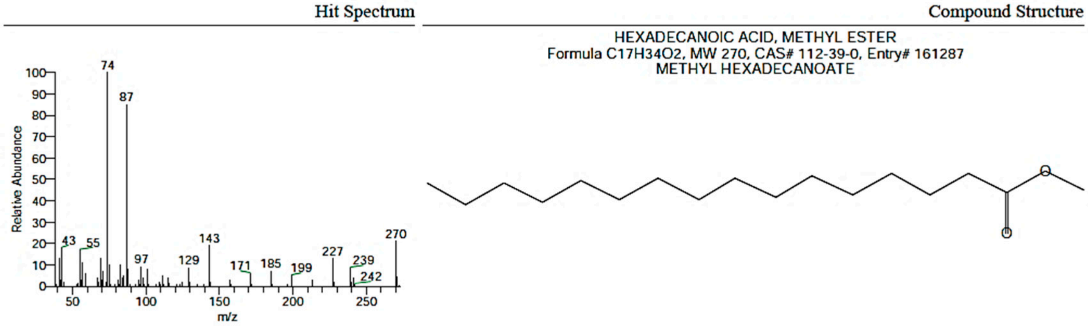Hit Spectrum<br>Compound Structure<br>HEXADECANOIC ACID, METHYL ESTER<br>Formula C17H34O2, MW 270, CAS# 112-39-0, Entry# 161287<br>METHYL HEXADECANOATE<br>Relative Abundance<br>m/z
7	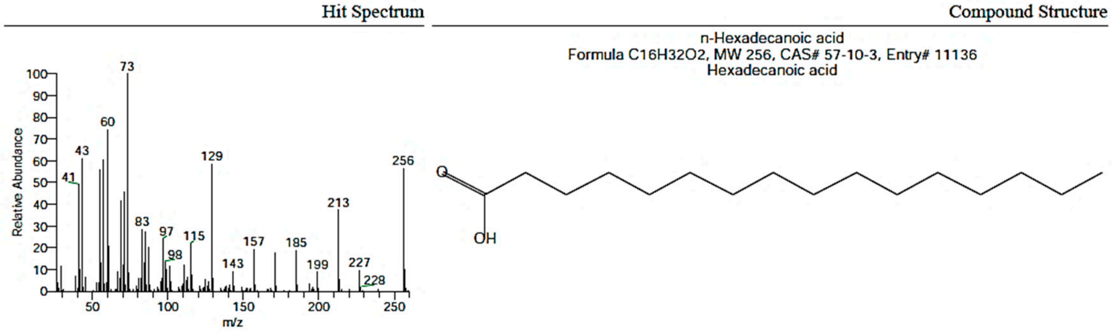Hit Spectrum<br>Compound Structure<br>n-Hexadecanoic acid<br>Formula C16H32O2, MW 256, CAS# 57-10-3, Entry# 11136<br>Hexadecanoic acid<br>Relative Abundance<br>m/z
8	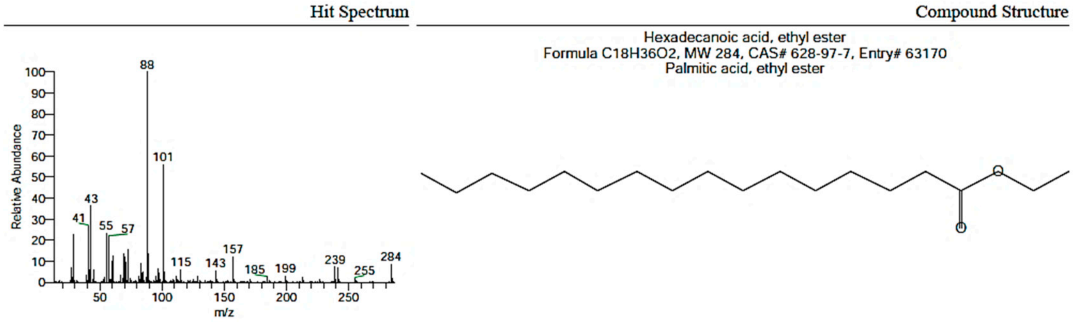Hit Spectrum<br>Compound Structure<br>Hexadecanoic acid, ethyl ester<br>Formula C18H36O2, MW 284, CAS# 628-97-7, Entry# 63170<br>Palmitic acid, ethyl ester<br>Relative Abundance<br>m/z

9

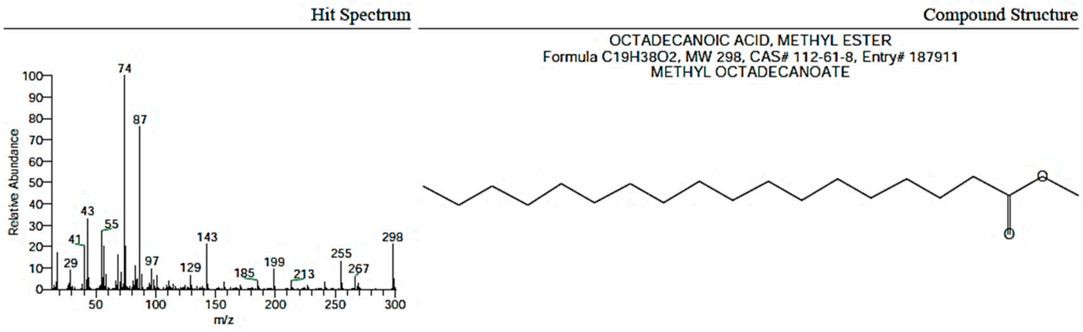
Hit Spectrum
Compound Structure
OCTADECANOIC ACID, METHYL ESTER
Formula C19H38O2, MW 298, CAS# 112-61-8, Entry# 187911
METHYL OCTADECANOATE
Relative Abundance
m/z

10

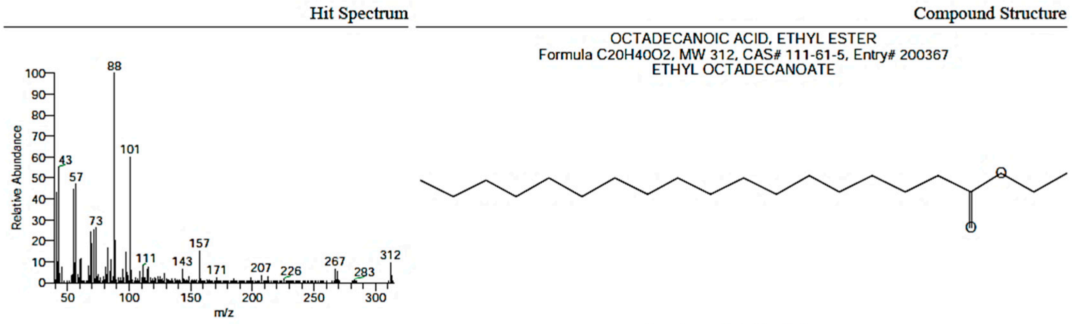
Hit Spectrum
Compound Structure
OCTADECANOIC ACID, ETHYL ESTER
Formula C20H40O2, MW 312, CAS# 111-61-5, Entry# 200367
ETHYL OCTADECANOATE
Relative Abundance
m/z

11

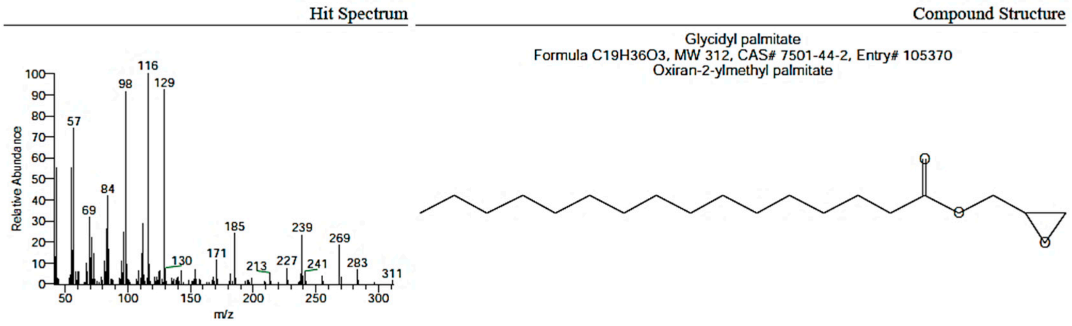
Hit Spectrum
Compound Structure
Glycidyl palmitate
Formula C19H36O3, MW 312, CAS# 7501-44-2, Entry# 105370
Oxiran-2-ylmethyl palmitate
Relative Abundance
m/z

12

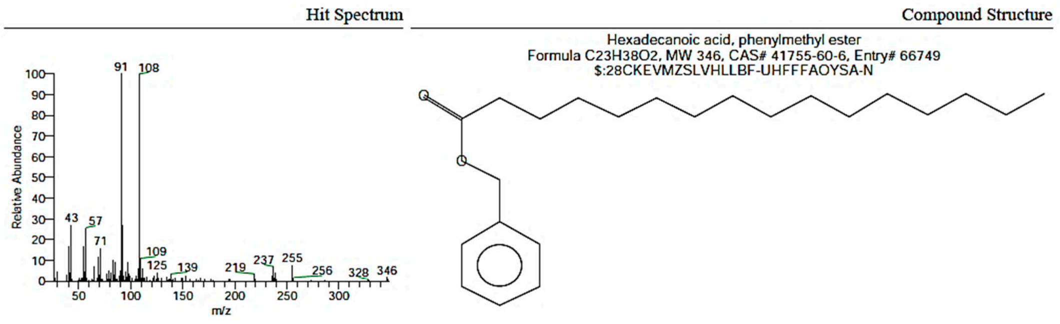
Hit Spectrum
Compound Structure
Hexadecanoic acid, phenylmethyl ester
Formula C23H38O2, MW 346, CAS# 41755-60-6, Entry# 66749
$:28CKEVMZSLVHLLBF-UHFFFAOYSA-N
Relative Abundance
m/z

Table S1. LC-MS/MS analysis of the ethanolic extract of *H. dicksoniae* in both negative and positive ionization modes.

Positive ion mode compounds (MS)	Retention Time (min)	Precursor m.z	Error PPM	Adduct	Reference m.z	Formula	MS/MS fragments	Ontology
4-aminophenol	1.038283	110.0085	-0.9	$[M^{+}H]^{+}$	110.06004	C_6H_7NO	110, 109, 73, 68, 67	Aniline and substituted anilines
Nicotinamide	1.12145	122.924	0	$[M^{+}H]^{+}$	123.05529	$C_6H_6N_2O$	122	Nicotinamides
L-Arginine	1.12145	175.1188	0.5	$[M^{+}H]^{+}$	175.11896	$C_6H_{14}N_4O_2$	158, 116, 84, 70, 60	L-alpha-amino acids
Pipecolate	1.322783	130.0862	0.8	$[M^{+}H]^{+}$	130.08626	$C_6H_{11}NO_2$	121, 119, 94, 66, 55, 53	Alpha amino acids
Adenine	1.322783	136.0616	0.4	$[M^{+}H]^{+}$	136.06177	$C_5H_5N_5$	103, 84, 70, 67, 56, 54	6-aminopurines
Choline	1.335117	104.1069	-0.3	$[M]^{+}$	104.10645	$C_5H_{14}NO$	87, 60, 59, 58	Cholines
L-Proline	1.335117	116.0708	-3.3	$[M^{+}H]^{+}$	116.0706	$C_5H_9NO_2$	98, 71, 69, 56, 53	Proline and derivatives
Pipecolate	1.377633	130.0496	0	$[M^{+}H]^{+}$	130.08626	$C_6H_{11}NO_2$	103, 84, 70, 57, 54	Alpha amino acids
Adenine	1.470433	136.0618	0.2	$[M^{+}H]^{+}$	136.06177	$C_5H_5N_5$	120, 109, 94, 97, 65, 53	6-aminopurines
L-Norleucine	1.688933	132.1017	-0.7	$[M^{+}H]^{+}$	132.1019	$C_6H_{13}NO_2$	114, 87, 71, 57, 55	L-alpha-amino acids
Caffeic acid	5.155583	181.0496	-0.5	$[M^{+}H]^{+}$	181.04953	$C_9H_8O_4$	163, 128, 95, 68, 51	Hydroxycinnamic acids
Luteolin-3', 7-di-O-glucoside	5.655383	611.1607	0.1	$[M^{+}H]^{+}$	611.16064	$C_{27}H_{30}O_{16}$	593, 355, 287, 195, 73	Flavonoid-7-O-glycosides
Luteolin-6-C-glucoside	6.784333	449.1075	-0.4	$[M^{+}H]^{+}$	449.10785	$C_{21}H_{20}O_{11}$	432, 377, 181, 133, 99, 53	Flavonoid C-glycosides
Cyanidin-3-O-(2''-O-beta-glucopyranosyl-beta-glucopyranoside)	7.076483	611.1608	0	$[M]^{+}$	611.1601	$C_{27}H_{31}O_{16}$	578, 308, 287, 241, 145, 61	Anthocyanidin-3-O-glycosides
Apigenin 8-C-glucoside	7.44895	433.1126	0.4	$[M^{+}H]^{+}$	433.11292	$C_{21}H_{20}O_{10}$	431, 413, 325, 282, 185, 92, 53	Flavonoid 8-C-glycosides
Cyanidin-3-glucoside	7.644283	449.107	0.3	$[M]^{+}$	449.1073	$C_{21}H_{21}O_{11}$	447, 311, 292, 288, 85, 61	Anthocyanidin-3-O-glycosides
Scopoletin	8.049933	193.0498	-1.4	$[M^{+}H]^{+}$	193.04953	$C_{10}H_8O_4$	192, 150, 123, 103, 76	7-hydroxycoumarins
Cholic acid	8.22365	409.1106	-1	$[M^{+}H]^{+}$	409.29486	$C_{24}H_{40}O_5$	407, 365, 271, 167, 160, 127	Trihydroxy bile acids, alcohols and derivatives
Peonidine-3-O-glucoside chloride	8.5199	463.1239	-0.3	$[M]^{+}$	463.12292	$C_{22}H_{23}O_{11}$	446, 301, 229, 147	Anthocyanidin-3-O-glycosides
Daphnetin	8.661333	179.1073	-3.6	$[M^{+}H]^{+}$	179.03389	$C_9H_6O_4$	178, 151, 132, 106, 84, 51	7,8-dihydroxycoumarins
3-Formylindole	8.82705	146.0599	0.2	$[M^{+}H]^{+}$	146.06004	C_9H_7NO	119, 116, 89, 64, 61, 50	Indoles
Luteolin	10.11582	287.0549	0.7	$[M^{+}H]^{+}$	287.05502	$C_{15}H_{10}O_6$	284, 200, 151, 125, 78, 50	Flavones
3,5,7-trihydroxy-4′-methoxyflavone	11.49525	301.0707	0.9	$[M^{+}H]^{+}$	301.07068	$C_{16}H_{12}O_6$	288, 229, 184, 126, 86, 67	Flavonols
Alpha-D-glucose-1-phosphate dipotassium salt dihydrate	13.1205	261.1024	0.4	$[M^{+}H]^{+}$	261.03699	$C_6H_{13}O_9P$	260, 215, 158, 115, 90, 51	Monosaccharide phosphates

Cholic acid	15.7687	409.1631	-0.7	$[M^+H]^+$	409.29486	$C_{24}H_{40}O_5$	407, 391	Trihydroxy bile acids, alcohols, and derivatives
Inosine-5'-monophosphate	16.72152	349.1441	-0.8	$[M^+H]^+$	349.05438	$C_{10}H_{13}N_4O_8P$	348	Purine ribonucleoside monophosphates
3,5,7-trihydroxy-4′-methoxyflavone	19.22985	301.1405	1.3	$[M^+H]^+$	301.07068	$C_{16}H_{12}O_6$	283, 245, 189, 133, 129	Flavonols
cyanidin-3-O-rutinoside	20.99535	595.2543	-0.2	$[M]^+$	595.16522	$C_{27}H_{31}O_{15}$	577, 520, 436, 397, 323	Anthocyanidin-3-O-glycosides
Isoguvacine	23.3188	128.143	0.9	$[M^+H]^+$	128.0706	$C_6H_9NO_2$	126, 102, 81, 58, 53	Hydropyridines
**Negative ion mode compounds (MS)**	**RT (min)**	**Precursor m.z**	**Error PPM**	**Adduct**	**Reference m.z**	**Formula**	**MS/MS fragments**	**Ontology**
Melibiose	1.225533	341.1171	-0.4	$[M^-H]^-$	341.10895	$C_{12}H_{22}O_{11}$	179, 143, 113, 87, 71, 59, 55	O-glycosyl compounds
Luteolin-3', 7-di-O-glucoside	5.65215	609.1602	-0.4	$[M^-H]^-$	609.14612	$C_{27}H_{30}O_{16}$	607, 448, 355, 298, 257, 96	Flavonoid-7-O-glycosides
Kaempferol-7-neohesperidoside	5.7043	593.1652	-0.4	$[M^-H]^-$	593.15118	$C_{27}H_{30}O_{15}$	575, 471, 383, 237, 101, 71	Flavonoid-7-O-glycosides
Quercitrin	5.716467	447.063	-0.1	$[M^-H]^-$	447.09329	$C_{21}H_{20}O_{11}$	445, 285, 195, 113, 87, 57	Flavonoid-3-O-glycosides
Myricitrin	5.728467	463.0583	0.6	$[M^-H]^-$	463.0882	$C_{21}H_{20}O_{12}$	297, 96, 95, 74	Flavonoid-3-O-glycosides
Myricitrin	5.728467	463.0585	0.2	$[M^-H]^-$	463.0882	$C_{21}H_{20}O_{12}$	297, 96, 95, 74	Flavonoid-3-O-glycosides
1-O-b-D-glucopyranosyl sinapate	6.267467	385.1226	0	$[M^-H]^-$	385.11401	$C_{17}H_{22}O_{10}$	383, 295, 175, 85, 55	Hydroxycinnamic acid glycosides
Caffeic acid	6.368433	179.0393	-0.5	$[M^-H]^-$	179.03499	$C_9H_8O_4$	161, 117, 106, 89, 65	Hydroxycinnamic acids
Luteolin-3', 7-di-O-glucoside	6.4306	609.161	-1.1	$[M^-H]^-$	609.14612	$C_{27}H_{30}O_{16}$	607, 563, 429, 327, 284, 151	Flavonoid-7-O-glycosides
Luteolin-6-C-glucoside	6.775583	447.1038	-0.8	$[M^-H]^-$	447.09329	$C_{21}H_{20}O_{11}$	445, 330, 228, 157, 93, 55	Flavonoid C-glycosides
Apigenin 8-C-glucoside	7.43905	431.1086	0.2	$[M^-H]^-$	431.09836	$C_{21}H_{20}O_{10}$	429, 305, 268, 175, 61	Flavonoid 8-C-glycosides
Maritimetin-6-O-glucoside	7.634533	447.1032	-0.7	$[M^-H]^-$	447.09329	$C_{21}H_{20}O_{11}$	445, 287, 201, 149, 131, 107	Aurone O-glycosides
Cyanidin-3-O-galactoside	8.154516	447.1032	0.5	$[M^-2H]^-$	447.09274	$C_{21}H_{21}O_{11}$	445, 325, 284, 227, 125	Anthocyanidin-3-O-glycosides
Luteolin-3', 7-di-O-glucoside	8.246667	609.1387	0.9	$[M^-H]^-$	609.14612	$C_{27}H_{30}O_{16}$	607, 591, 429, 393, 179, 135	Flavonoid-7-O-glycosides
Maritimetin-6-O-glucoside	8.3185	447.1035	-0.7	$[M^-H]^-$	447.09329	$C_{21}H_{20}O_{11}$	445, 327, 217, 151, 107, 65	Aurone O-glycosides
Kaempferol-3-glucuronide	8.51415	461.1194	0.3	$[M^-H]^-$	461.07254	$C_{21}H_{18}O_{12}$	459, 399, 356, 285, 255	Flavonoid-3-O-glucuronides
(+)-3,3′,4′,5,7-Pentahydroxyflavan	9.040466	289.1187	-0.9	$[M^-H]^-$	289.07175	$C_{15}H_{14}O_6$	243, 137, 97, 57	Catechins
Luteolin	10.11092	285.0468	-0.5	$[M^-H]^-$	285.04047	$C_{15}H_{10}O_6$	181, 155, 134, 107, 89, 51	Flavones
3,5,7-trihydroxy-4′-methoxyflavone	11.48617	299.0633	-0.2	$[M^-H]^-$	299.05612	$C_{16}H_{12}O_6$	284, 256, 216, 159, 63	Flavonols
Acacetin	12.62352	283.1987	-1	$[M^-H]^-$	283.06119	$C_{16}H_{12}O_5$	281, 203, 145, 117, 57	4'-O-methylated flavonoids
Hesperetin	15.77862	301.1649	-0.8	$[M^-H]^-$	301.07175	$C_{16}H_{14}O_6$	265, 247, 203, 195, 164	4'-O-methylated flavonoids
Gamma-linolenic acid	21.01952	277.2239	-0.2	$[M^-H]^-$	277.21732	$C_{18}H_{30}O_2$	276, 219, 155, 83, 57	Linoleic acid and derivatives
L-Threonic acid hemicalcium salt	22.10142	134.8981	-2.8	$[M^-H]^-$	135.02989	$C_4H_8O_5$	117, 107, 99, 93, 92	Sugar acids and derivatives

Table S2. Binding affinity (ΔG, kcal/mol)–based interaction strength between all GC-MS-identified ligands and target proteins.

**Ligands** / **Target proteins**	**Anticancer**			**Antioxidant**			**Antimicrobial**							
	**CA2**	**PPARA**	**PRKCE**	**FABP2**	**FABP3**	**FABP4**	***C. albicans***				***B. subtilis***	***S. aureus***	***P. vulgaris***	***E. coli***
							**ERG2**	**ERG3**	**ERG5**	**ERG11**	**dacC**	**pbp**	**rpsA**	**rpsQ**
2(3H)-furanone, 5-heptyldihydro	-5.0	-5.8	-5.5	-6.8	-5.4	-5.4	-6.3	-5.5	-5.8	-5.4	-5.1	-5.1	-4.2	-3.6
1,2-benzenedicarboxylic acid, diethyl ester	-4.9	-6.2	-5.8	-6.5	-6.0	-5.8	-7.0	-6.5	-5.5	-6.1	-5.3	-5.3	-4.9	-4.0
1-(4-isopropylphenyl)-2-methylpropyl acetate	-5.2	-6.4	-6.8	-7.5	-6.5	**-6.9**	-8.0	**-7.3**	-6.5	-6.7	-6.0	-6.5	**-5.3**	-4.5
Benzoic acid, phenylmethyl ester	**-6.0**	-6.8	**-7.1**	-8.2	**-7.0**	-6.8	**-8.3**	-7.0	**-7.9**	**-7.2**	**-6.1**	**-6.6**	-5.0	**-4.9**
Isopropyl myristate	-4.3	-5.8	-5.4	-7.0	-5.5	-5.7	-6.5	-6.1	-6.2	-5.9	-4.5	-4.9	-3.4	-3.1
Hexadecanoic acid, methyl ester	-4.5	-5.8	-5.4	-6.8	-5.9	-5.8	-6.2	-6.1	-5.9	-5.8	-5.2	-5.3	-4.0	-3.3
n-Hexadecanoic acid	-4.5	-5.6	-5.4	-6.9	-5.7	-5.6	-6.2	-6.0	-6.0	-5.4	-5.2	-5.1	-3.6	-3.1
Hexadecanoic acid, ethyl ester	-4.5	-5.7	-5.1	-7.2	-5.5	-5.8	-6.2	-6.3	-6.1	-5.4	-4.7	-5.3	-3.6	-4.0
Octadecanoic acid, methyl ester	-4.2	-6.1	-5.4	-7.2	-5.6	-5.8	-6.6	-6.2	-6.0	-5.7	-4.8	-5.2	-3.7	-3.1
Octadecanoic acid, ethyl ester	-4.2	-6.1	-5.5	-7.1	-5.7	-5.8	-6.4	-6.4	-6.2	-5.6	-5.2	-5.1	-3.8	-3.5
Glycidyl palmitate	-4.3	-6.3	-5.5	-7.0	-5.7	-6.1	-6.3	-6.6	-6.2	-5.6	-5.1	-5.7	-3.8	-3.6
Hexadecanoic acid, phenylmethyl ester	-5.3	**-7.4**	-5.9	**-8.5**	-6.5	-6.4	-7.8	-7.1	-6.7	-6.4	-5.3	-5.4	-4.2	-3.3

Bold values indicate the highest binding affinity (i.e., the most negative ΔG values), reflecting strong interactions between the ligand and the active site of the target protein.

Table S3. Molecular docking analysis showing 2D and 3D binding interactions between GC-MS–identified ligands and target proteins with the highest binding affinity (i.e., the most negative ΔG values).

**Dock score (kcal/mol) of ligand-protein interaction**	**2D binding Interaction**	**3D binding Interaction**
Benzoic acid, phenylmethyl ester with CA2 (ΔG = -6.0 kcal/mol)	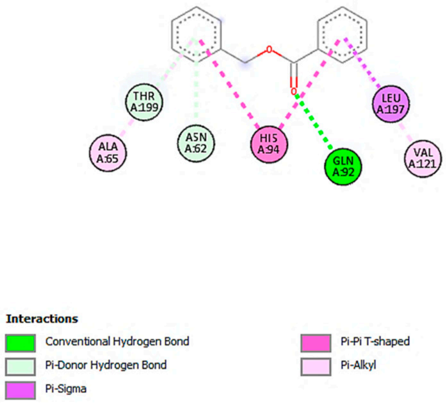	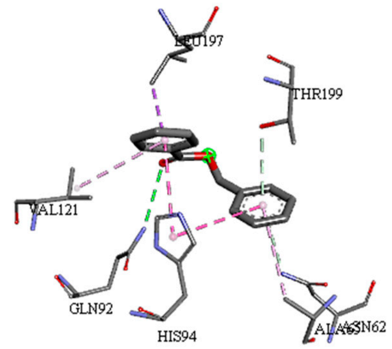
Hexadecanoic acid, phenylmethyl ester with PPARA (ΔG = -7.4 kcal/mol)	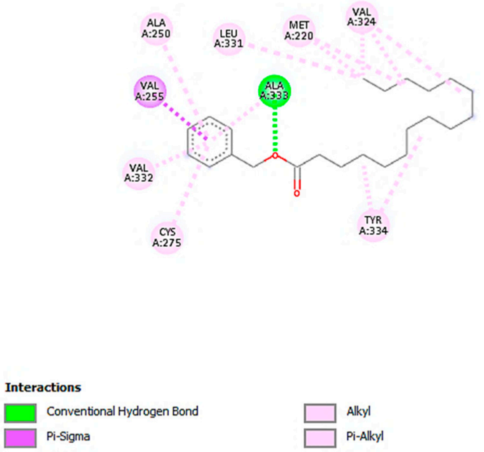	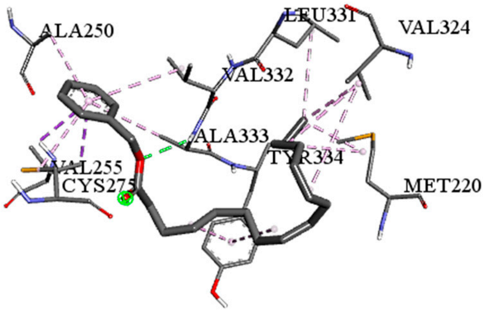
Benzoic acid, phenylmethyl ester with PRKCE (ΔG = -7.1 kcal/mol)	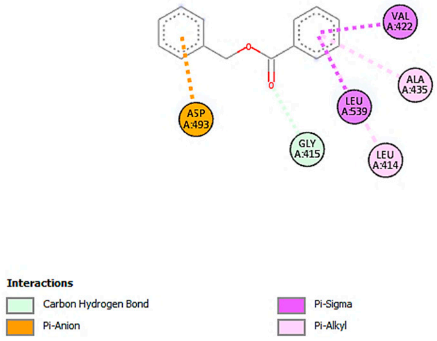	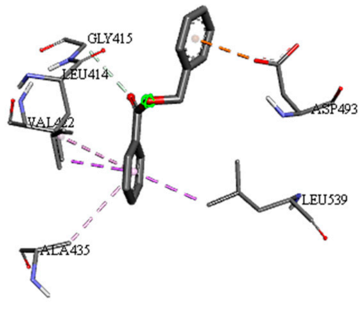

<table>
<tr>
<td>Hexadecanoic acid, phenylmethyl ester with FABP2
(ΔG = -8.5 kcal/mol)</td>
<td>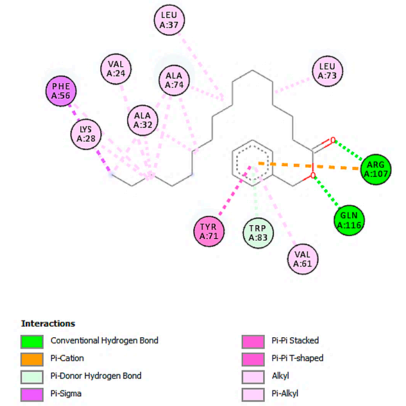
</td>
<td>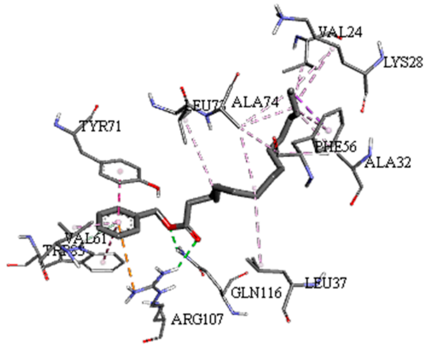
</td>
</tr>
<tr>
<td>Benzoic acid, phenylmethyl ester with FABP3
(ΔG = -7.0 kcal/mol)</td>
<td>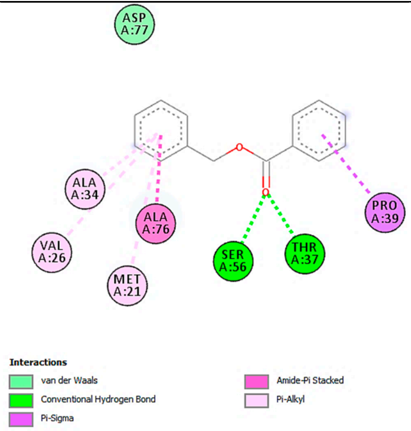
</td>
<td>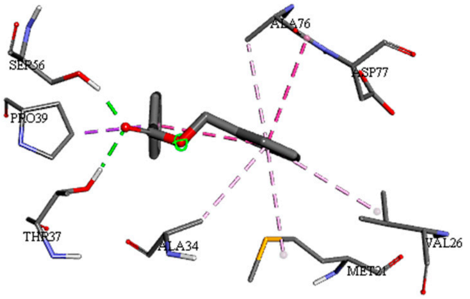
</td>
</tr>
<tr>
<td>1-(4-isopropylphenyl)-2 -methylpropyl acetate with FABP4
(ΔG = -6.9 kcal/mol)</td>
<td>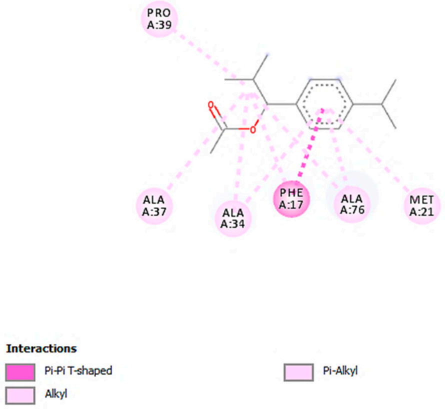
</td>
<td>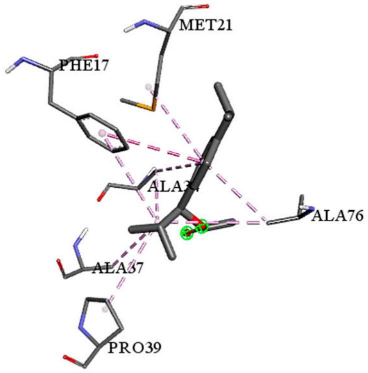
</td>
</tr>
</table>

<table>
<tr>
<td>Benzoic acid, phenylmethyl ester with ERG2-C. albicans
(ΔG = -8.3 kcal/mol)</td>
<td>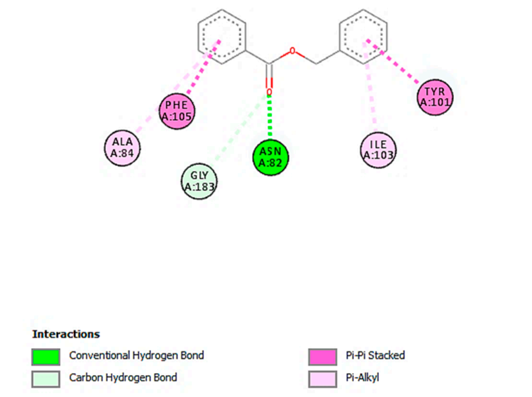
</td>
<td>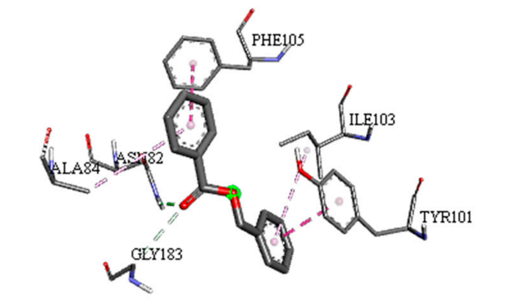
</td>
</tr>
<tr>
<td>1-(4-isopropylphenyl)-2 -methylpropyl acetate with ERG3-C. albicans
(ΔG = -7.3 kcal/mol)</td>
<td>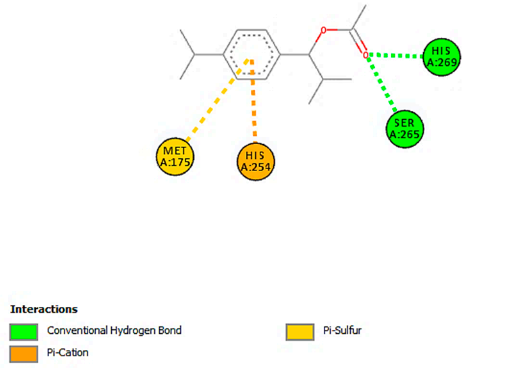
</td>
<td>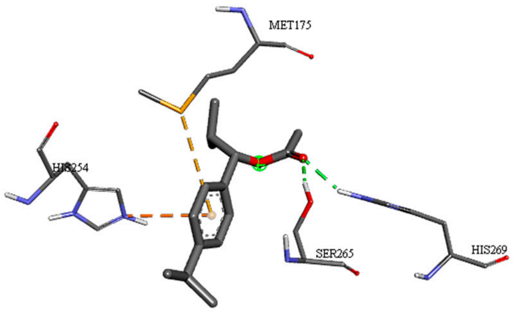
</td>
</tr>
<tr>
<td>Benzoic acid, phenylmethyl ester with ERG5-C. albicans
(ΔG = -7.9 kcal/mol)</td>
<td>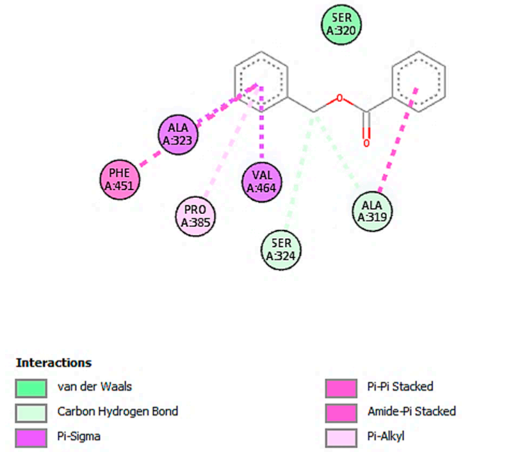
</td>
<td>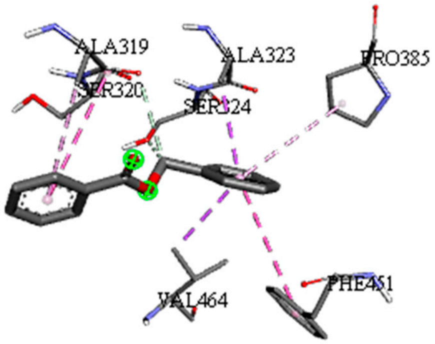
</td>
</tr>
</table>

Benzoic acid, phenylmethyl ester with ERG11-*C. albicans* (ΔG = -7.2 kcal/mol)	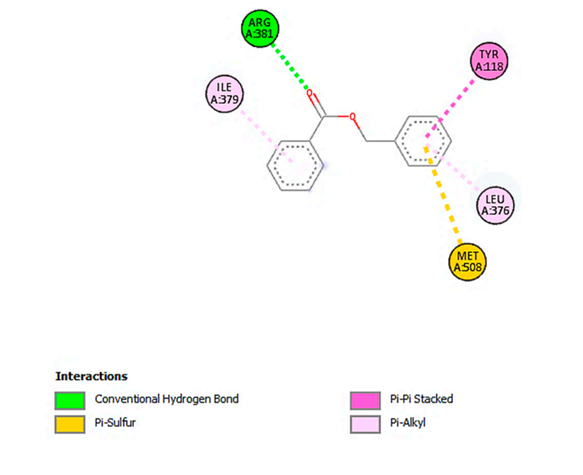	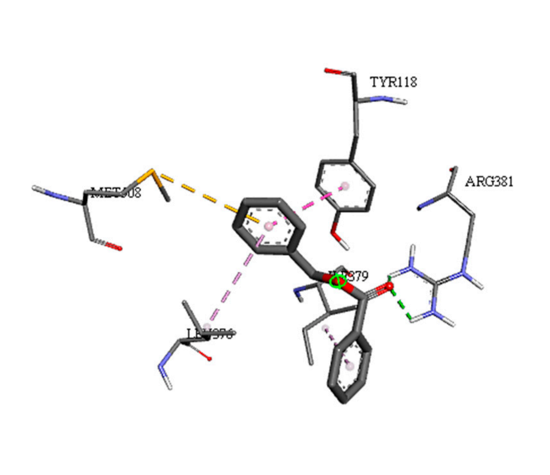
Benzoic acid, phenylmethyl ester with dacC-*B. subtilis* (ΔG = -6.1 kcal/mol)	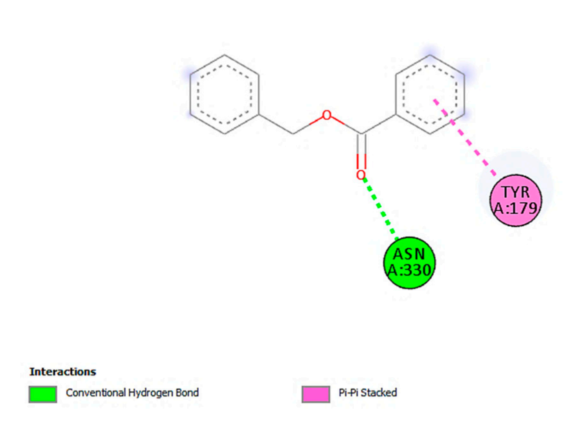	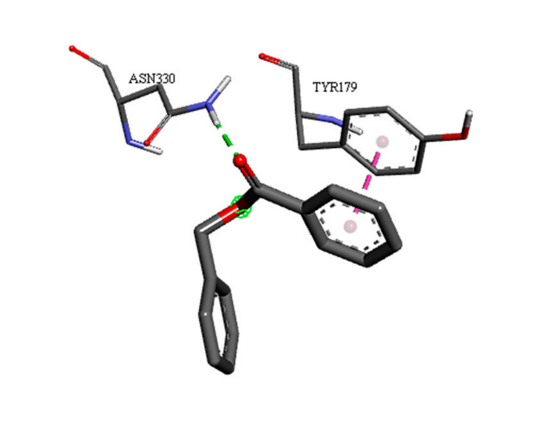
Benzoic acid, phenylmethyl ester with pbp-*S. aureus* (ΔG = -6.6 kcal/mol)	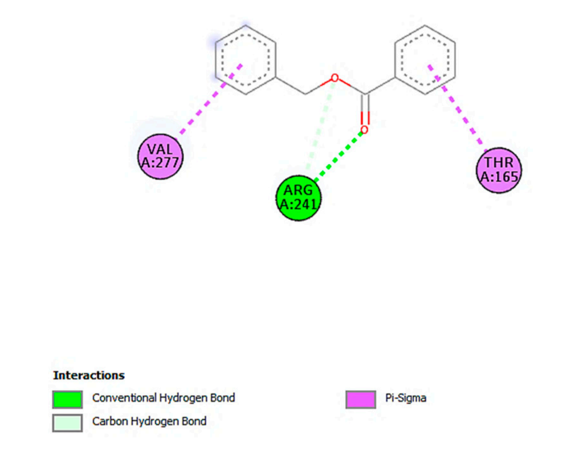	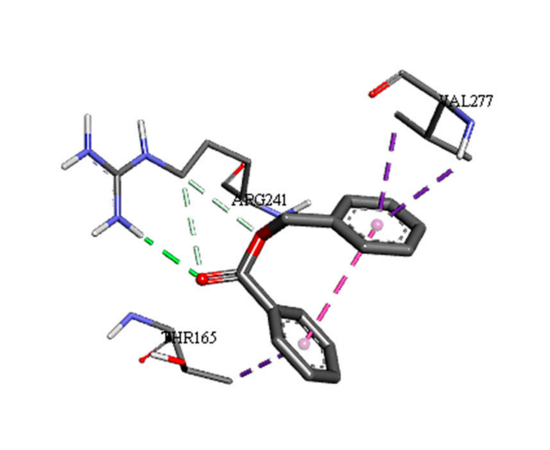

1-(4-isopropylphenyl)-2 -methylpropyl acetate with rpsA-*P. vulgaris*<br>(ΔG = -5.3 kcal/mol)	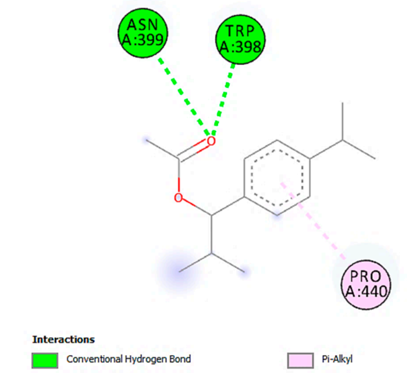	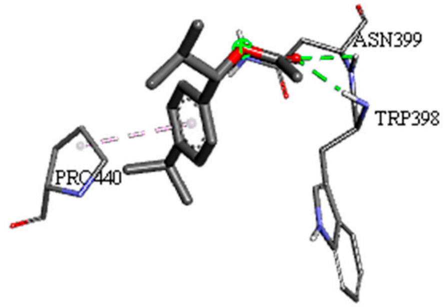
Benzoic acid, phenylmethyl ester with rpsQ-*E. coli*<br>(ΔG = -4.9 kcal/mol)	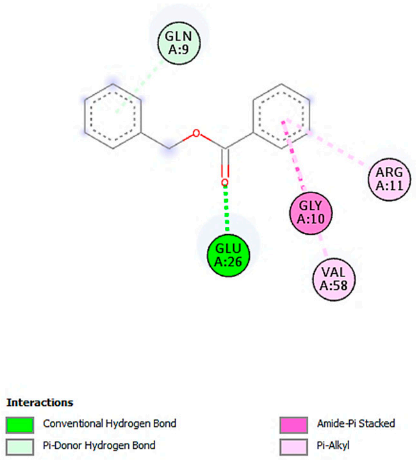	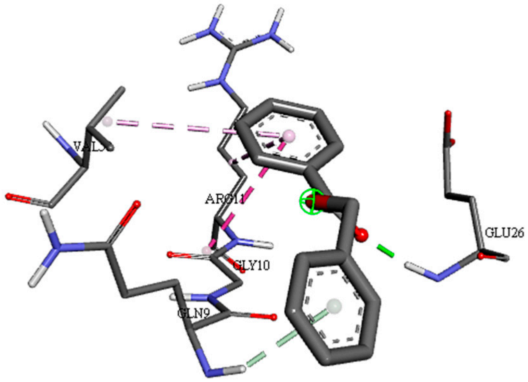

Table S4. Binding affinity (ΔG, kcal/mol)–based interaction strength between all the LC-MS/MS–identified ligands and target proteins.

Ligands \ Target proteins	Anticancer			Antioxidant			Antimicrobial							
							C. albicans				*B. subtilis*	*S. aureus*	*P. vulgaris*	*E. coli*
	**CA2**	**PPARA**	**PRKCE**	**FABP2**	**FABP3**	**FABP4**	**ERG2**	**ERG3**	**ERG5**	**ERG11**	**dacC**	**pbp**	**rpsA**	**rpsQ**
Negative Ion Mode Compounds (MS)														
(+)-3,3′,4′,5,7-pentahydroxyflavan	-6.3	-7.3	-7.9	-8.6	-8.2	-8.3	-8.8	-7.9	-7.4	-8.1	-7.4	-7.8	-6.9	-5.9
1-O-b-D-glucopyranosyl sinapate	-6.0	-7.8	-7.6	-8.9	-8.6	-8.5	-6.1	-6.5	-7.0	-7.3	-7.2	-7.5	-6.4	-5.4
Acacetin	-6.3	-7.0	-8.0	-8.2	-7.8	-8.1	**-9.2**	**-8.7**	-8.1	-7.9	-7.5	-8.1	-6.5	-5.9
Gamma-linolenic acid	-4.9	-6.6	-5.5	-6.6	-6.2	-6.2	-6.6	-6.8	-5.5	-5.8	-4.3	-4.9	-4.0	-3.0
Hesperetin	-6.5	-7.5	-8.6	-8.5	-8.3	-8.3	-8.9	**-8.7**	-7.7	-7.8	-7.8	-8.3	-6.7	-6.0
Kaempferol-3-glucuronide	-6.8	-8.3	-8.4	**-9.3**	-9.2	-9.5	-7.0	-6.5	-8.8	-8.7	-7.7	-7.8	-6.6	-5.6
Kaempferol-7-neohesperidoside	**-8.0**	-7.8	**-9.8**	-9.1	-6.5	-9.4	-8.0	-6.6	-9.4	-9.1	-9.0	-9.0	-7.3	-6.4
L-Threonic acid hemicalcium salt	-4.1	-4.7	-4.3	-5.0	-4.6	-4.7	-4.2	-4.9	-4.4	-4.5	-5.1	-4.5	-4.0	-3.9
Maritimetin-6-O-glucoside	-6.6	-8.0	-9.1	-6.2	-6.2	-5.7	-7.9	-6.3	-8.2	-8.6	-7.6	-8.7	-6.7	-6.1
Melibiose	-5.5	-6.9	-6.7	-7.5	-7.5	-7.6	-7.7	-7.9	-6.7	-7.0	-6.9	-6.9	-5.7	-5.2
Myricitrin	-6.9	-7.5	-9.7	-8.9	-9.4	-9.6	-6.7	-6.9	-8.5	-9.5	-8.0	-8.3	-6.5	-6.3
Quercitrin	-6.9	-7.6	-9.4	-8.5	-9.5	-9.7	-6.8	-6.2	-8.9	-9.4	-8.2	-8.1	-6.5	-6.4
Positive Ion Mode Compounds (MS)														
3,5,7-trihydroxy-4′-methoxyflavone	-6.6	-7.3	-8.4	-8.3	-7.9	-8.2	-9.1	**-8.7**	-8.4	-7.7	-7.6	-8.0	-6.7	-5.8
3-Formylindole	-4.9	-6.0	-5.8	-7.3	-5.9	-5.6	-6.8	-6.0	-5.8	-5.8	-5.4	-5.2	-4.5	-4.4
Adenine	-5.3	-5.8	-5.1	-5.6	-5.1	-5.4	-5.5	-5.5	-5.5	-5.3	-5.0	-5.4	-4.5	-4.3
Alpha-D-glucose-1-phosphate dipotassium salt dihydrate	-4.9	-6.8	-5.8	-6.5	-5.9	-6.2	-6.4	-6.6	-5.7	-6.6	-5.7	-5.6	-4.9	-4.7
Apigenin 8-C-glucoside	-6.6	-7.3	-8.7	-9.1	**-9.9**	-9.5	-8.0	-6.6	-8.5	-8.5	-8.0	-8.1	-6.5	-6.2
Caffeic acid	-5.5	-6.7	-6.0	-7.0	-6.2	-6.0	-6.5	-6.5	-6.0	-6.6	-6.3	-5.8	-5.1	-4.8
Cholic acid	-6.7	-6.8	-8.3	-6.4	-8.5	-8.9	-8.9	-6.9	-8.2	-8.4	-7.5	-8.4	-6.1	-5.8
Choline	-3.2	-3.8	-3.2	-3.4	-3.3	-3.6	-3.6	-3.5	-3.6	-3.5	-3.3	-3.5	-2.8	-2.8
Cyanidin-3-glucoside	-6.9	-8.0	-8.8	-8.7	-9.1	-9.6	-7.4	-7.3	-8.4	-8.4	-8.9	-8.2	-6.7	-6.0

Cyanidin-3-O-(2''-O-beta-glucopyranosyl-beta-glucopyranoside)	-7.1	-7.4	-8.8	-6.6	-6.0	-5.9	-6.5	-6.0	-9.6	-9.3	-8.2	-8.7	-6.6	-6.2
Cyanidin-3-O-rutinoside	-7	-8.6	-8.5	-7.1	-9.1	**-10.4**	-7.1	-6.5	-9.2	-9.1	**-9.2**	-8.7	-6.9	**-7.2**
Daphnetin	-5.4	-7.2	-6.9	-7.8	-6.4	-6.9	-7.1	-6.8	-6.7	-6.5	-6.4	-6.3	-5.4	-4.7
Inosine-5'-monophosphate	-6.4	-7.3	-7.0	-8.5	-7.5	-7.6	-8.1	-8.6	-7.0	-7.1	-6.8	-7.1	-6.1	-5.6
Isoguvacine	-4.5	-5.1	-4.8	-5.3	-5.2	-4.8	-5.5	-5.3	-5.0	-4.9	-4.7	-4.8	-4.1	-4.0
Luteolin	-6.5	-8.0	-8.4	-8.6	-8.7	-8.4	-9.0	-8.3	-8.1	-7.7	-7.8	-8.0	-6.8	-5.8
Luteolin-3',7-di-O-glucoside	-7.2	**-9.2**	-8.9	-7.0	-6.9	-9.2	-7.3	-6.9	**-9.7**	**-9.6**	-8.7	**-9.6**	**-7.9**	-6.6
Luteolin-6-C-glucoside	-6.6	-8.1	-8.6	-6.2	-6.4	-5.9	-7.7	-6.9	-8.8	-8.2	-7.8	-8.7	-7.3	-6.4
L-Arginine	-4.8	-6.1	-4.9	-5.5	-5.0	-5.4	-5.8	-5.9	-5.0	-5.0	-5.7	-4.6	-4.5	-3.9
L-Norleucine	-3.9	-4.9	-4.2	-5.3	-4.3	-4.5	-4.8	-4.9	-4.4	-4.4	-4.8	-4.4	-3.6	-3.5
L-Proline	-4	-4.9	-4.3	-5.1	-4.3	-4.7	-4.6	-4.8	-4.8	-4.6	-4.4	-4.7	-3.8	-3.7
Nicotinamide	-4.6	-5.0	-4.7	-6.0	-4.9	-5.0	-5.2	-5.5	-5.0	-5.4	-4.9	-5.0	-4.1	-3.9
Peonidine-3-O-glucoside chloride	-6.7	-7.3	-8.5	-8.6	-9.0	-9.7	-7.3	-6.9	-8.4	-8.6	-8.6	-8.1	-6.7	-6.0
Pipecolate	-4.6	-5.2	-4.7	-5.9	-4.9	-4.9	-5.2	-5.1	-5.1	-5.2	-5.0	-4.9	-4.0	-3.9
4-Aminophenol	-4.6	-4.9	-4.4	-5.2	-4.7	-4.4	-5.0	-4.8	-4.7	-4.8	-4.5	-4.7	-4.1	-4.0
Scopoletin	-5.1	-6.5	-6.7	-7.1	-6.3	-6.3	-7.2	-6.9	-6.5	-6.7	-6.5	-6.3	-5.2	-4.7

Bold values indicate the highest binding affinity (i.e., the most negative ΔG values), reflecting strong interactions between the ligand and the active site of the target protein.

Table S5. Molecular docking analysis showing 2D and 3D binding interactions between LC-MS/MS–identified ligands and target proteins with the highest binding affinity (i.e., the most negative ΔG values).

**Dock score (kcal/mol) of ligand-protein interaction**	**2D binding Interaction**	**3D binding Interaction**
Kaempferol-7-neohesperidoside with CA2<br><br>(ΔG = -8.0 kcal/mol)	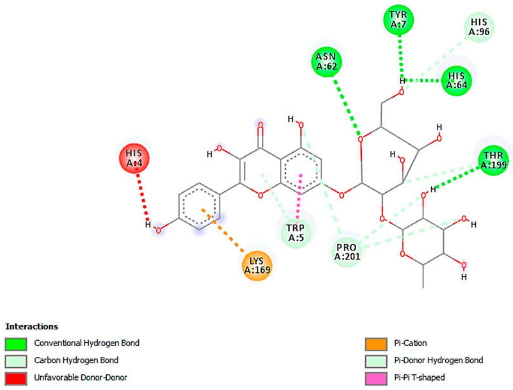	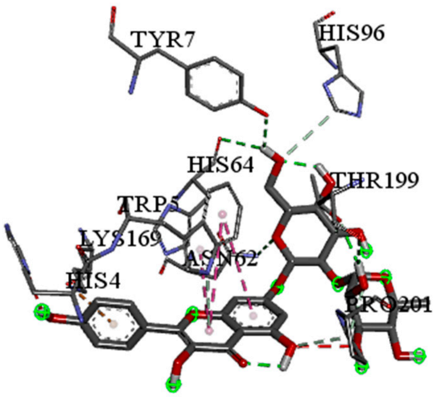
Luteolin-3', 7-di-O-glucoside with PPARA<br><br>(ΔG = -9.2 kcal/mol)	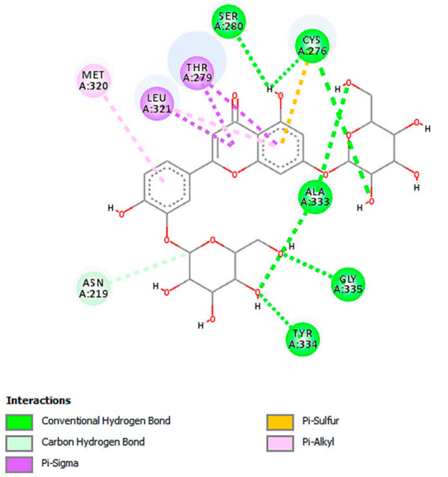	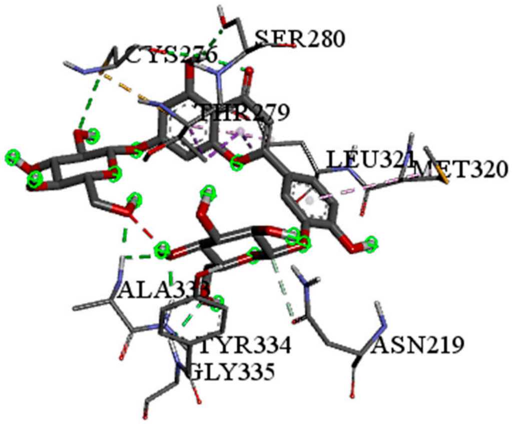
Kaempferol-7-neohesperidoside with PRKCE<br><br>(ΔG = -9.8 kcal/mol)	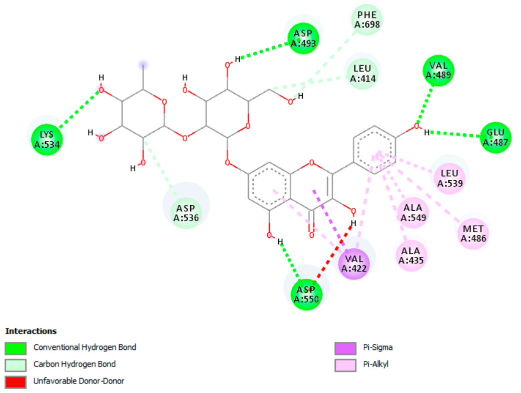	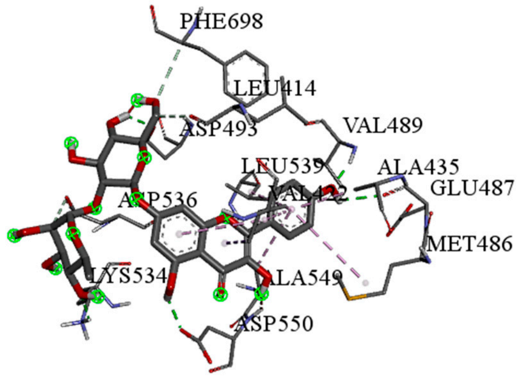

<table>
<tr><td>Kaempferol-3-glucuronide with FABP2

(ΔG = -9.3 kcal/mol)</td><td>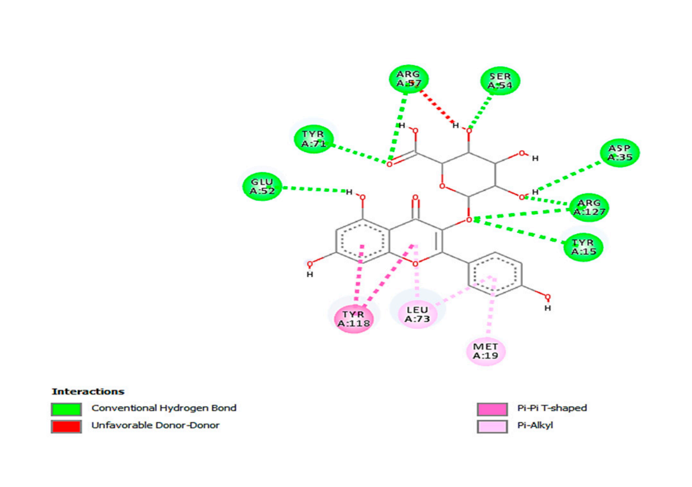
</td><td>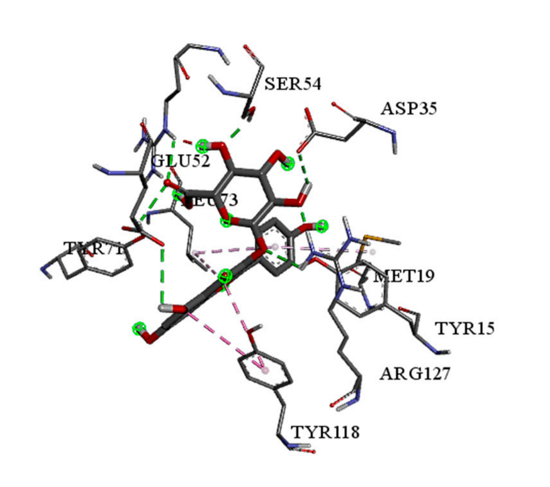
</td></tr>
<tr><td>Apigenin 8-C-glucoside with FABP3

(ΔG = -9.9 kcal/mol)</td><td>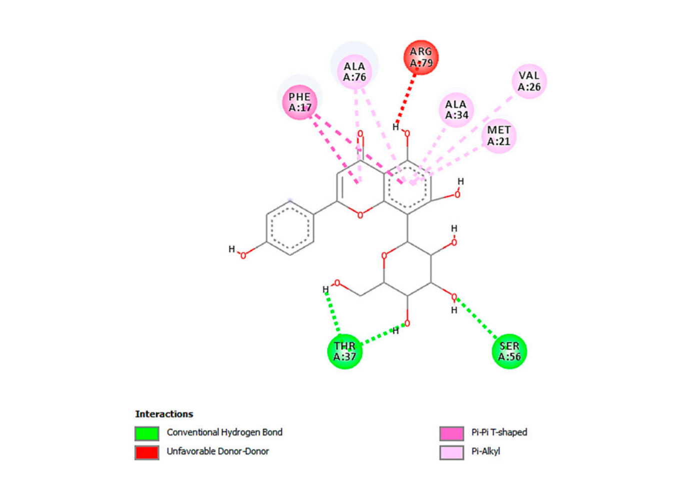
</td><td>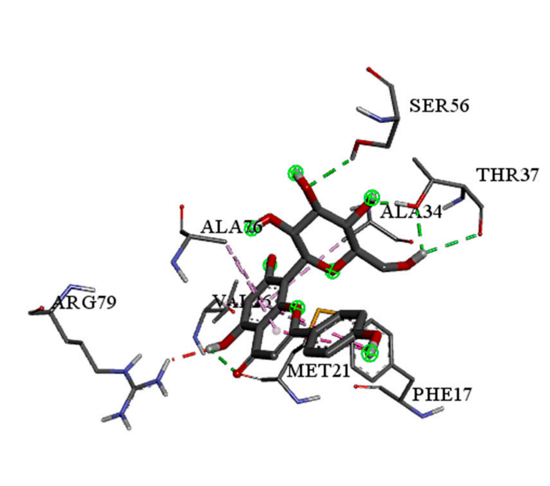
</td></tr>
<tr><td>Cyanidin-3-O-rutinoside with FABP4

(ΔG = -10.4 kcal/mol)</td><td>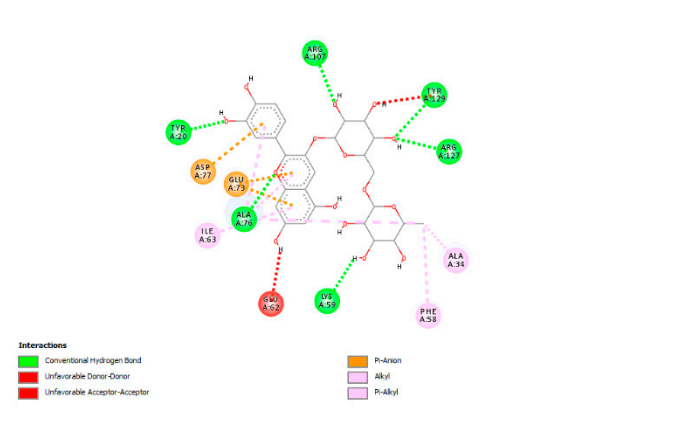
</td><td>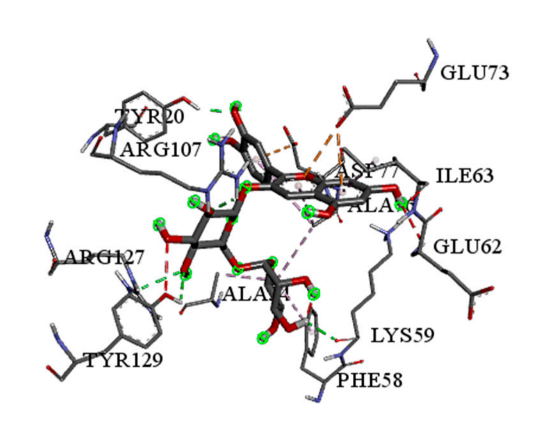
</td></tr>
<tr><td>Acacetin with ERG2-C. albicans

(ΔG = -9.2 kcal/mol)</td><td>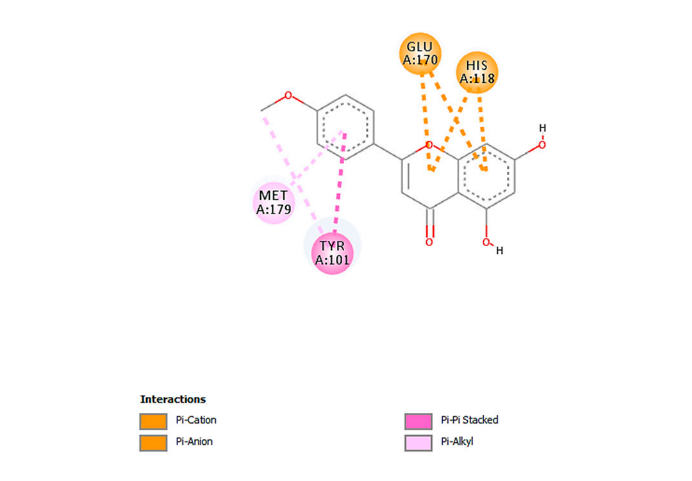
</td><td>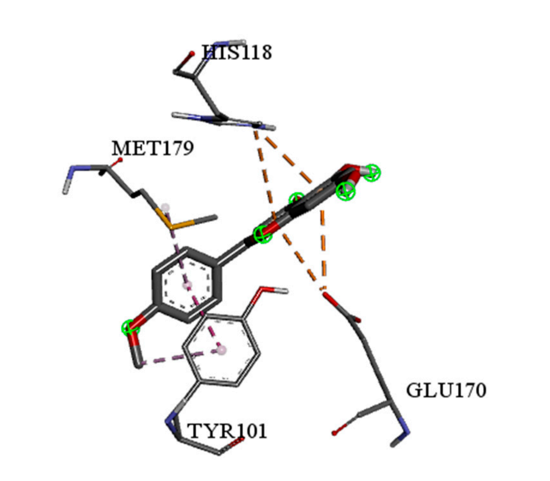
</td></tr>
</table>

<table>
<tr><td>Acacetin with ERG3-C. albicans

(ΔG = -8.7 kcal/mol)</td><td>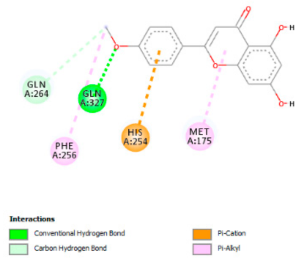
</td><td>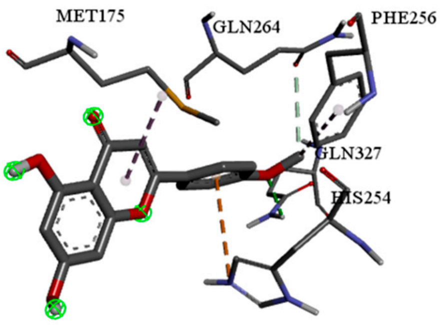
</td></tr>
<tr><td>Hesperetin with ERG3-C. albicans

(ΔG = -8.7 kcal/mol)</td><td>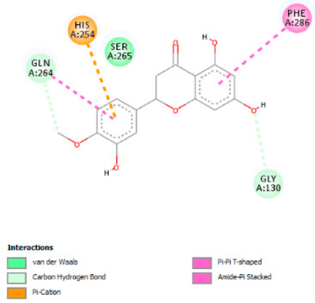
</td><td>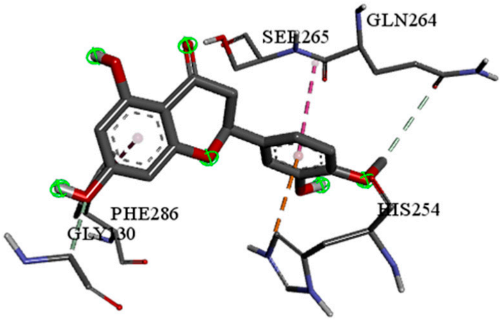
</td></tr>
<tr><td>3,5,7-trihydroxy-4′-methoxyflavone with ERG3-C. albicans

(ΔG = -8.7 kcal/mol)</td><td>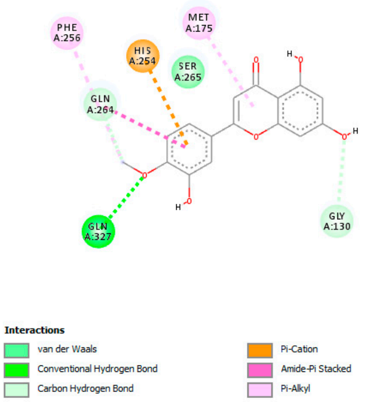
</td><td>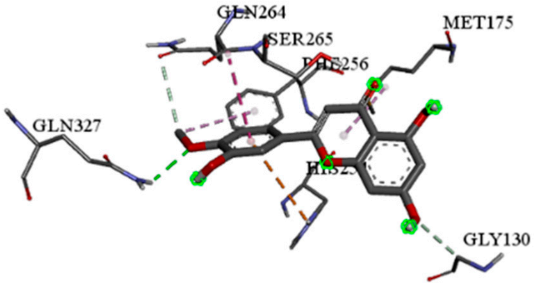
</td></tr>
<tr><td>Luteolin-3', 7-di-O-glucoside with ERG5-C. albicans

(ΔG = -9.7 kcal/mol)</td><td>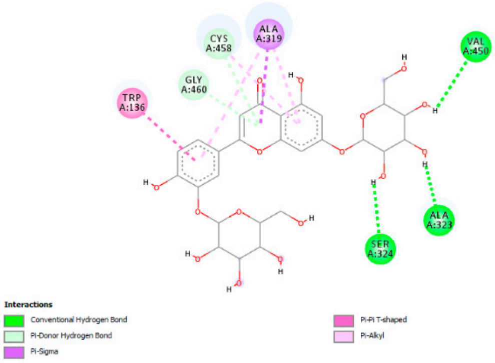
</td><td>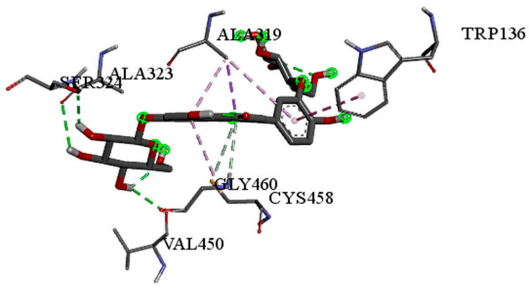
</td></tr>
</table>

<table>
<tr>
<td>Luteolin-3', 7-di-O-glucoside with ERG11-C. albicans

(ΔG = -9.6 kcal/mol)</td>
<td>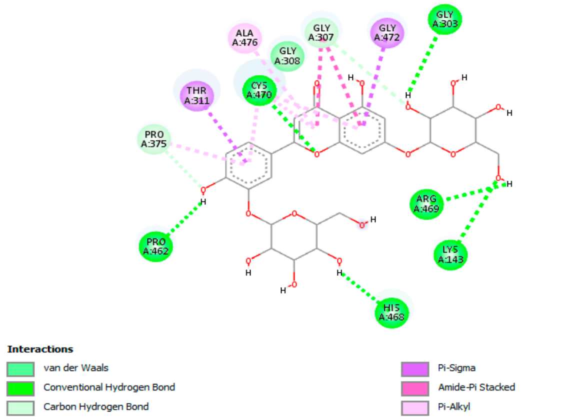
</td>
<td>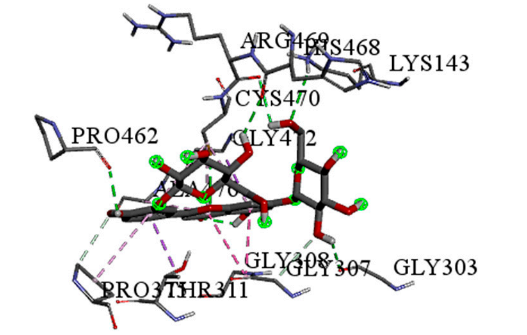
</td>
</tr>
<tr>
<td>Cyanidin-3-O-rutinoside with dacC-B. subtilis

(ΔG = -9.2 kcal/mol)</td>
<td>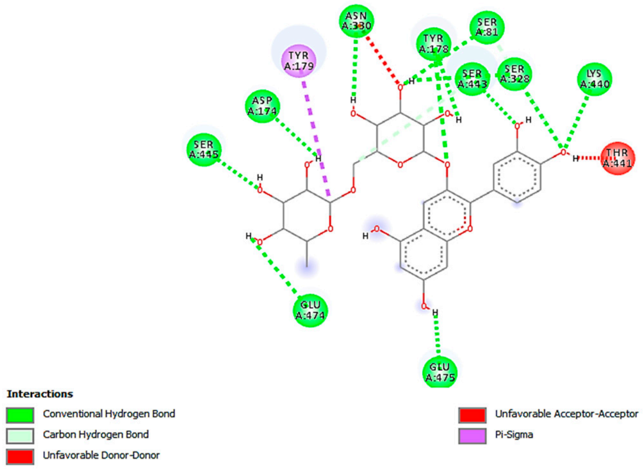
</td>
<td>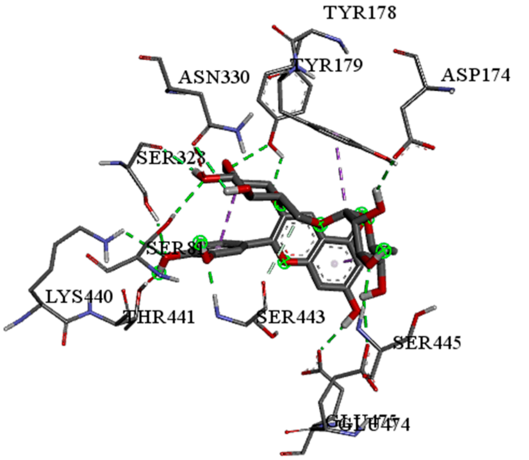
</td>
</tr>
<tr>
<td>Luteolin-3', 7-di-O-glucoside with pbp-S. aureus

(ΔG = -9.6 kcal/mol)</td>
<td>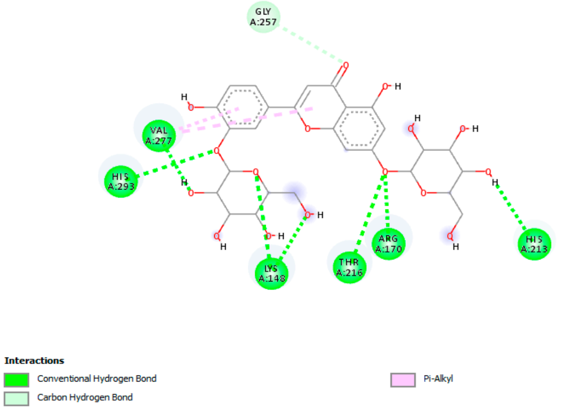
</td>
<td>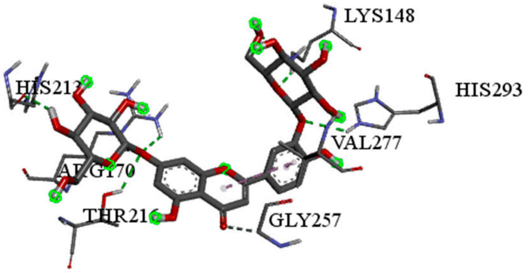
</td>
</tr>
</table>

Luteolin-3', 7-di-O-glucoside with rpsA-*P. vulgaris*<br><br>(ΔG = -7.9 kcal/mol)	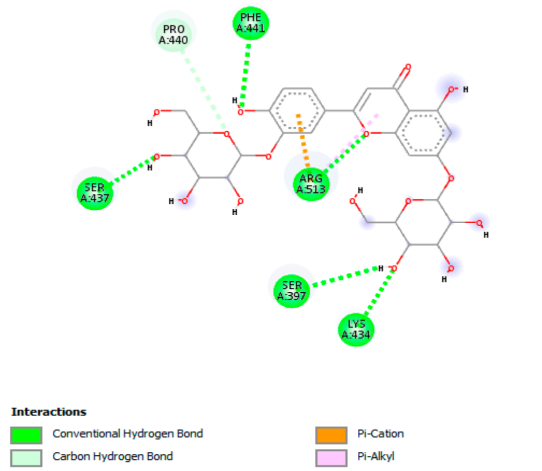	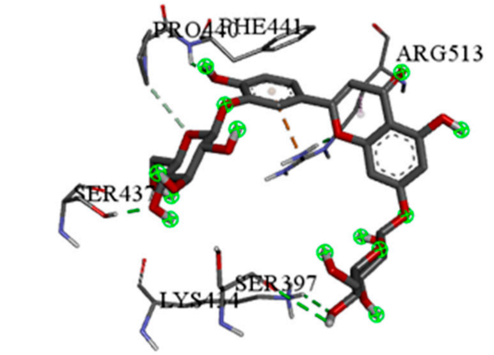
Cyanidin-3-O-rutinoside with rpsQ-*E. coli*<br><br>(ΔG = -7.2 kcal/mol)	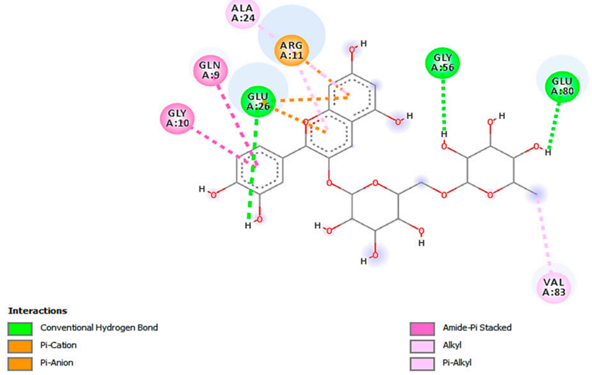	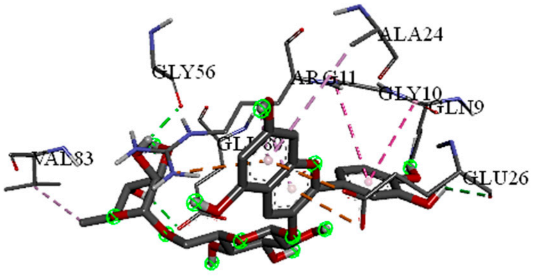
